# The Placenta Regulates Intrauterine Fetal Growth via Exosomal PPARγ

**DOI:** 10.1002/advs.202404983

**Published:** 2025-02-14

**Authors:** Xiaofang Luo, Biao Huang, Ping Xu, Hao Wang, Baozhen Zhang, Li Lin, Jiujiang Liao, Mingyu Hu, Xiyao Liu, Jiayu Huang, Yong Fu, Mark D. Kilby, Rodney E. Kellems, Xiujun Fan, Yang Xia, Philip N. Baker, Hongbo Qi, Chao Tong

**Affiliations:** ^1^ Reproductive Medicine Center The First Affiliated Hospital of Chongqing Medical University Chongqing 400016 China; ^2^ State Key Laboratory of Maternal and Fetal Medicine of Chongqing Municipality The First Affiliated Hospital of Chongqing Medical University Chongqing 400016 China; ^3^ Ministry of Education International Collaborative Laboratory of Reproduction and Development Chongqing 400016 China; ^4^ Department of Obstetrics Women and Children's Hospital of Chongqing Medical University Chongqing 401147 China; ^5^ Department of Biochemistry & Molecular Biology University of Texas McGovern Medical School at Houston Houston TX 77030 USA; ^6^ Institute of Metabolism and System Research University of Birmingham, and the Fetal Medicine Centre Birmingham Women's and Children's Foundation Trust Edgbaston B15 2TT UK; ^7^ Shenzhen Institutes of Advanced Technology Chinese Academy of Sciences Shenzhen Guangdong 518055 China; ^8^ College of Life Sciences University of Leicester Leicester LE1 7RH UK; ^9^ National Clinical Research Center for Child Health and Disorders Ministry of Education Key Laboratory of Child Development and Disorders Children's Hospital of Chongqing Medical University Chongqing 401122 China

**Keywords:** adipogenesis, exosomes, fetal growth restriction, placentas, PPARγ, trophoblasts

## Abstract

Abnormal adipogenesis is a major contributor to fetal growth restriction (FGR) and its associated complications. However, the underlying etiology remains unclear. Here, it is reported that the placentas of women with pregnancies complicated with FGR exhibit peroxisome proliferator‐activated receptor γ (PPARγ) inactivation. In mice, trophoblast‐specific ablation of murine PPARγ reproduces the phenotype of human fetuses with FGR and defective adipogenesis. Coculture of trophoblasts with preadipocytes significantly improves preadipocyte commitment and differentiation and increases the transcription of a series of adipogenic genes via intercellular transfer of exosomal PPARγ proteins. Moreover, nanoparticle‐mediated placenta‐specific delivery of rosiglitazone (RGZ) significantly rescues adipogenesis defects in an FGR‐induced mouse model. In summary, the placenta is a major reservoir of PPARγ. An insufficient supply of placental PPARγ to fetal preadipocytes via exosomes during late gestation is a major mechanism underlying FGR. Preclinically, placenta‐targeted RGZ administration can be a promising interventional therapy for FGR and/or defective intrauterine fat development.

## Graphical abstract

The placenta expresses much higher levels of PPARγ than fetal adipose tissue. Trophoblastic exosomes transport PPARγ into fetal preadipocytes via fetoplacental circulation and thus regulate adipogenic genes distinct from endogenous PPARγ. Compromised placental PPARγ leads to fetal adipogenesis deficiency and intrauterine growth restriction, reversible by placenta‐targeted PPARγ activation.

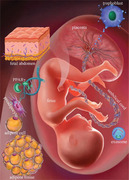

## 1 Introduction

Babies that do not attain their genetic growth potential in utero are considered to have fetal growth restriction (FGR).^[^ ^1^ ^]^ FGR is often identified on the basis of the concept of ‘small for dates’, defined as the birth weight (BW) below the tenth percentile for gestational age.^[^ ^2^ ^]^ Infants with FGR often have a reduced fetal growth velocity in utero^[^ ^3^ ^]^ and an increased risk for various adverse perinatal outcomes.^[^ ^1^ ^]^ FGR followed by accelerated postnatal growth in early life leads to adipose tissue dysfunction and insulin resistance, which increases the risk of type 2 diabetes mellitus (T2DM) and metabolic syndrome in these offspring later in life.^[^ ^4^ ^]^ Studies have shown that the subcutaneous fat mass is significantly decreased in growth‐restricted fetuses, which is the major difference between fetuses with growth restriction and those with normal growth.^[^ ^5^, ^6^ ^]^

Peroxisome proliferator‐activated receptor γ (PPARγ) is a member of the nuclear transcription receptor superfamily^[^ ^7^ ^]^ that directly binds to target genes as an asymmetric heterodimer with retinoid X receptors (RXRs) after activation by ligands.^[^ ^8^ ^]^ This molecule is highly expressed in adipocytes and plays an essential role in controlling adipogenesis, lipid metabolism, and insulin sensitivity.^[^ ^9^, ^10^ ^]^ In addition, PPARγ is expressed in placental tissue and has been shown to be critical for appropriate placental function and fetal growth. In mice, PPARγ‐deficient trophoblast stem cells have a limited capacity to differentiate into labyrinthine trophoblasts.^[^ ^11^ ^]^ Furthermore, PPARγ‐deficient mice exhibited abnormal vascularization at the earliest developmental stage, terminal epithelial differentiation of trophoblasts, and deficits in lipid accumulation in the placenta.^[^ ^12^ ^]^ Global deletion of PPARγ in mice led to embryonic lethality, yet PPARγ‐null mice survive to term following preservation of placental PPARγ via tetraploid rescue.^[^ ^12^, ^13^ ^]^ Accordingly, pharmacological inhibition of PPARγ during the latter half of pregnancy‐induced FGR in rats.^[^ ^14^ ^]^ Although current evidence has revealed the involvement of placental PPARγ in the modulation of fetal growth during pregnancy, mainly through placental development, the direct regulation of fetal adipogenesis by placental PPARγ remains to be elucidated.

In this study, we determined the underlying mechanism and transport pathway of placental PPARγ in the regulation of in utero adipogenesis. Our results revealed the therapeutic potential of targeting placental PPARγ for FGR management.

## 2 Results

### 2.1 Placental PPARγ Expression Coincides with Fetal Adipogenesis

**Figure 1:**
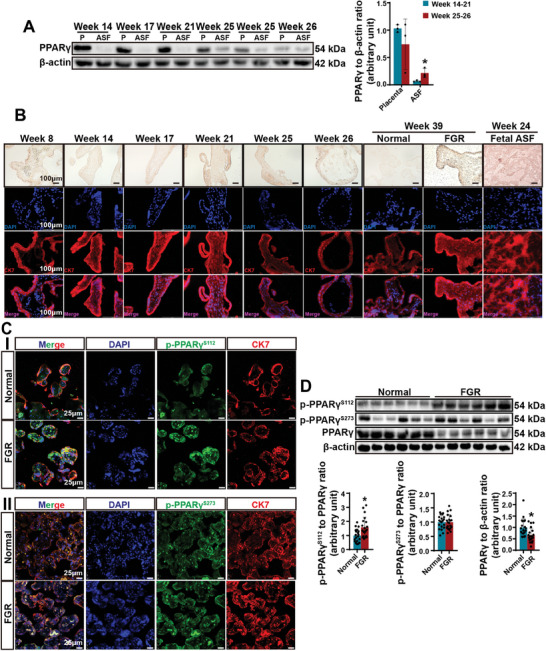
The placenta expresses high levels of PPARγ, but placental PPARγ expression is compromised in FGR. A) Immunoblot analysis of PPARγ in human placenta and fetal ASF samples collected at the indicated gestational weeks. *n* = 1 biological sample per group. Student's *t*‐test. *P < 0.05. B) Oil red O staining and immunostaining of CK7 and Perilipin 1 in human placenta and fetal ASF sections collected from women with normal pregnancies and women with FGR‐complicated pregnancies at the indicated gestational weeks. *n* = 2 biological samples per group; one representative image from each group is shown. Scale bar: 100 µm. C) Immunostaining for p‐PPARγ^S112^ (I) and p‐PPARγ^S273^ (II) in sections of human normal and FGR‐affected term placentas. *n* = 20 biological samples per group; one representative image from each group is shown. Scale bar: 25 µm. D) Immunoblot analysis of p‐PPARγ^S112^, p‐PPARγ^S273^, and PPARγ in normal human and FGR‐affected placentas. *n *= 20 biological samples per group; data for six representative samples per group are shown. Student's *t*‐test. *P < 0.05.

PPARγ is expressed in human villi beginning at the sixth week of pregnancy, but its expression substantially increases after the placenta develops, peaking at 14 weeks of gestation, and then gradually decreases until the end of gestation (Figure S1A, Supporting Information). Intriguingly, placentas presented much greater PPARγ protein expression than did fetal abdominal subcutaneous fat (ASF) tissues at 14 weeks of gestation (**Figure** **1**A). In contrast, PPARγ levels in fetal ASF were significantly greater than those in placentas at 25 weeks of gestation (Figure 1A). Similarly, the mouse placenta presented high PPARγ expression compared with that in the fetus, with fetal PPARγ levels gradually increasing during late gestation (Figure S1B,C, Supporting Information). However, the abundance of PPARγ did not result in substantial lipid synthesis in the placenta (Figure 1B). Thus, the inverse change in the expression of PPARγ in the placenta and fetus suggests that the placenta may serve as a PPARγ reservoir to supply the fetus, beginning in mid–late gestation to support adipogenesis.

### 2.2 PPARγ Expression is Compromised in FGR‐Affected Placentas

Upon ligand binding, the modulation of PPARγ phosphorylation determines its specific activities. Dephosphorylation of Ser273 is essential for the insulin‐sensitizing activity of PPARγ,^[^ ^15^ ^]^ whereas dephosphorylation of Ser112 is essential for its proadipocytic transcriptional activity.^[^ ^16^ ^]^ Although p‐PPARγ^S112^ and p‐PPARγ^S273^ are the predominant forms in trophoblasts in the human placenta (Figure 1C), we observed that the level of p‐PPARγ^S112^ was significantly increased but that of p‐PPARγ^S273^ was not different in the FGR‐affected placentas compared with the normal placentas (Figure 1D). In addition, the levels of other adipogenic factors, including C/EBPα, the SREBP1 precursor, and mature SREBP1,^[^ ^17^, ^18^ ^]^ were unaltered in the FGR‐affected placentas (Figure S1D, Supporting Information). The phosphorylation of acetyl‐CoA carboxylase (ACC) and the expression of PGC‐1α were suppressed, and the expression of ACC and SIRT1 was maintained in the FGR‐affected placentas (Figure S1E,F, Supporting Information), suggesting that PPARγ in the placenta is likely capable of modulating adipogenesis in the fetus but not of regulating placental fatty acid catabolism in situ.

**Figure 2:**
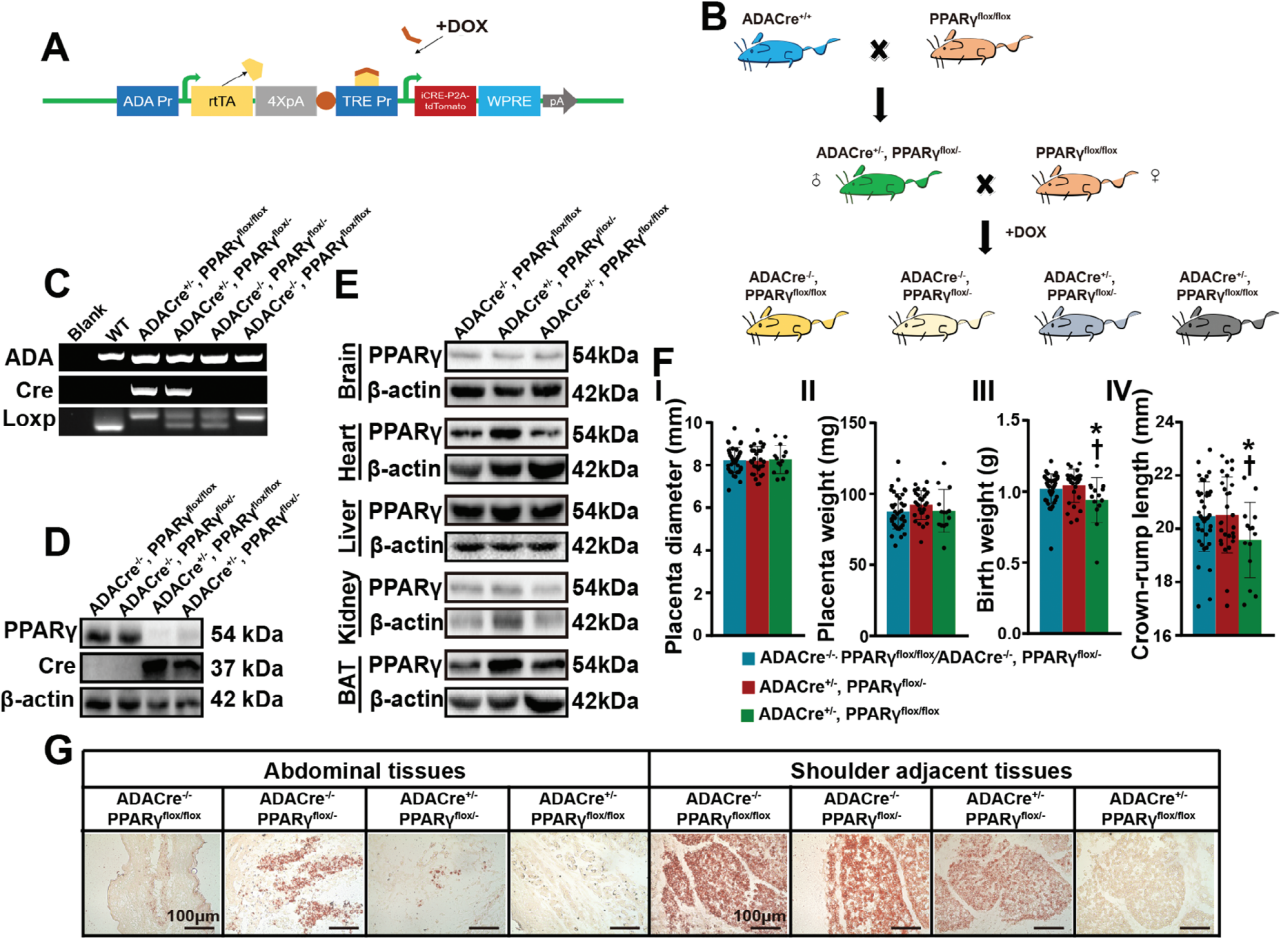
Placenta‐specific PPARγ ablation results in reduced amounts of adipose tissue and FGR. A) Component diagram of inducible ADA‐Cre expression in mice controlled by the Tet‐on system. B) Mating procedure for the *ADACre^+/+^* mice and *PPARγ^flox/flox^* mice. C) Genotyping analysis of the ADA gene, Cre recombinase transgene, and loxP transgene in mouse fetuses of the indicated genotypes via PCR. D) Immunoblot analysis of PPARγ and Cre recombinase in the placentas of mice of the indicated genotypes at GD 18.5. E) Immunoblot analysis of PPARγ in the brain, heart, liver, kidney, and BAT of fetuses of the indicated genotypes at GD 18.5. F) Analysis of placental diameter (I), placental weight (II), fetal birth weight (III), and CRL (IV) in the indicated groups at GD 18.5. *ADACre^−/−^, PPARγ^flox/flox^/ADACre^−/−^, PPARγ^flox/−^: n *= 38 litters from 12 dams. *ADACre^+/−^, PPARγ^flox/−^: n* = 28 litters from 11 dams. *ADACre^+/−^, PPARγ^floxflox^: n* = 14 litters from 10 dams. One‐way ANOVA. *P < 0.05 versus A*DACre^−/−^, PPARγ^flox/flox^/ADACre^−/−^, PPARγ^flox/−^*. †*P* < 0.05 versus *ADACre^+/−^, PPARγ^flox/−^*. G) Oil red O‐stained sections of abdominal tissue and shoulder‐adjacent tissue from mouse fetuses of the indicated genotypes. *n* = 6 litters from 3 dams in each group; one representative image from each group is shown. Scale bar: 100 µm.

Previous studies have shown that global PPARγ gene knockout results in severe lipodystrophy and embryonic lethality but that these phenotypes can be rescued by supplementing PPARγ‐null embryos with wild‐type placentas via aggregation with tetraploid embryos.^[^ ^12^, ^13^ ^]^ Conversely, dramatically reduced subcutaneous fat masses have been observed in fetuses with FGR, and this characteristic constitutes the major difference between fetuses with growth restriction and those with normal growth.^[^ ^5^, ^6^ ^]^ Thus, to validate the pivotal role of placental PPARγ in regulating fetal growth, especially adipogenesis in utero, we established a mouse line with inducible placenta‐specific PPARγ knockout by using ADA‐Cre mice, with no effect on PPARγ expression in the fetal brain, heart, liver, kidney or brown adipose tissue (BAT)^[^ ^19^ ^]^ (**Figure** **2**A–E). Global placental PPARγ knockout resulted in FGR as well as impaired adipogenesis in both abdominal and shoulder‐adjacent tissues (Figure 2F,G), the typical manifestation of FGR, whereas doxycycline (DOX) treatment did not affect placental or fetal weight at birth in the wild‐type control group (Figure S1G, Supporting Information).

### 2.3 Trophoblast PPARγ Promotes 3T3‐L1 Cell Differentiation

**Figure 3:**
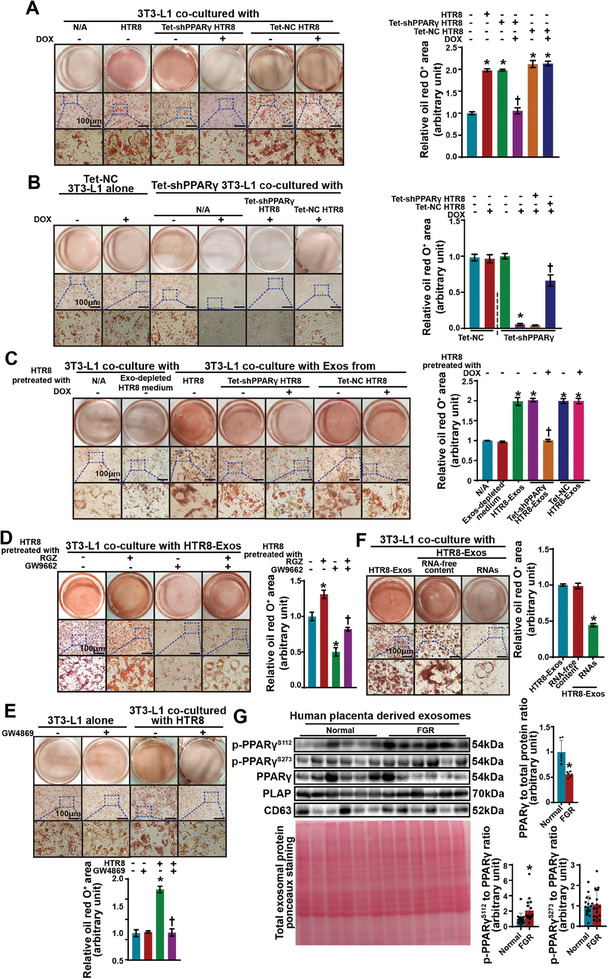
Trophoblast‐mediated preadipocyte differentiation is dependent on the transfer of exosomal PPARγ. A) Oil red O‐stained 3T3‐L1 cells on differentiation day 8 after coculture with no treatment (N/A), HTR8 cells, Tet‐shPPARγ HTR8 cells, or Tet‐NC HTR8 cells. *n* = 3 independent experiments. Two‐way ANOVA. *P < 0.05 versus N/A, †*P* < 0.05 versus. HTR8 cells. Scale bar: 100 µm. B) Oil red O staining of Tet‐NC 3T3‐L1 cells on differentiation day 8 and Tet‐shPPARγ 3T3‐L1 cells on differentiation day 8 after coculture with no treatment (N/A), Tet‐shPPARγ HTR8 cells, or Tet‐NC HTR8 cells. *n* = 3 independent experiments. Two‐way ANOVA. *P < 0.05 versus DOX‐untreated (DOX‐) Tet‐shPPARγ 3T3‐L1 cells, †*P *< 0.05 versus DOX+ Tet‐shPPARγ 3T3‐L1 cells. Scale bar: 100 µm. C) Oil red O‐stained 3T3‐L1 cells on differentiation day 8 after coculture with no treatment (N/A), Exo‐depleted HTR8 culture medium (the upper layer was centrifuged and free of exosomes when the exosomes were isolated), Exos derived from HTR8 cells, DOX‐pretreated/untreated Tet‐shPPARγ HTR8 cells, or Tet‐NC HTR8 cells were used. *n* = 3 independent experiments with 3 independent preparations of exosomes. Two‐way ANOVA. *P < 0.05 versus N/A. †*P* < 0.05 versus HTR8 cells. Scale bar: 100 µm. D) Oil red O‐stained 3T3‐L1 cells on differentiation day 8 after coculture with Exos from HTR8 cells pretreated with RGZ, GW9662, or both. *n* = 3 independent experiments with 3 independent preparations of exosomes. Two‐way ANOVA. *P < 0.05 vs. untreated cells, †*P* < 0.05 versus GW9662‐treated cells. Scale bar: 100 µm. E) Oil red O‐staining of 10 µm GW4869‐treated/untreated 3T3‐L1 cells on differentiation day 8 after coculture with/without HTR8 cells. GW4869 was applied at the beginning of culture and throughout differentiation. *n* = 3 independent experiments. Two‐way ANOVA. *: *P *< 0.05 versus untreated 3T3‐L1 cells. †*P *< 0.05 versus untreated cocultured 3T3‐L1 cells. Scale bar: 100 µm. F) Oil red O‐stained 3T3‐L1 cells on differentiation day 8 after coculture with whole Exos, exosomal RNAs, or exosomal RNA‐free content from HTR8 cells. *n* = 3 independent experiments with 3 independent preparations of exosomes. One‐way ANOVA. *P < 0.05 versus whole Exos. Scale bar: 100 µm. G) Immunoblot analysis of p‐PPARγ^S112^, p‐PPARγ^S273^, PPARγ, PLAP, and CD63 in placental exosomes from women with normal pregnancies and those with FGR‐complicated pregnancies. The loading amount of total protein was determined via Ponceau S staining. *n* = 17 biological samples per group; data for six representative samples per group are shown. Student's *t*‐test. *P < 0.05.

To determine whether placental trophoblasts play a role in the regulation of fetal adipogenesis, we cocultured 3T3‐L1 preadipocytes with various types of cells from the fetal–maternal interface (Figure S2A, Supporting Information). The differentiation of 3T3‐L1 cells was significantly increased only in the presence of HTR8 trophoblasts (Figure S2B, Supporting Information), which was consistent with the high expression of PPARγ in HTR8 cells (Figure S2C, Supporting Information). To assess the effects of trophoblast PPARγ on the regulation of preadipocyte differentiation, we generated trophoblasts with DOX‐inducible PPARγ knockdown (Tet‐shPPARγ HTR8 cells) and preadipocytes (Tet‐shPPARγ 3T3‐L1 cells) (Figure S2D–H, Supporting Information). The DOX‐treated Tet‐shPPARγ HTR8 (PPARγ‐KD) cells failed to increase the differentiation of 3T3‐L1 cells (**Figure** **3**A), demonstrating a crucial role for trophoblast PPARγ in the maturation of preadipocytes. Surprisingly, the defective differentiation of PPARγ‐KD 3T3‐L1 cells (DOX‐treated Tet‐shPPARγ 3T3‐L1 cells) due to PPARγ depletion was significantly rescued by coincubation with Tet‐NC HTR8 cells but not PPARγ‐KD HTR8 cells (Figure 3B). These findings indicate the critical involvement of PPARγ in trophoblastic support of preadipocyte differentiation when the amount of endogenous PPARγ in preadipocytes is limited. Moreover, PPARγ activity in HTR8 cells was altered by treatment with the PPARγ agonist rosiglitazone (RGZ) or the PPARγ antagonist GW9662 (Figure S2I,J, Supporting Information). PPARγ activation in HTR8 cells further stimulated lipogenesis in PPARγ‐KD 3T3‐L1 cells, whereas PPARγ inhibition abolished the effects of HTR8 cells on 3T3‐L1 cell differentiation (Figure S2K, Supporting Information). These findings suggest that it might be feasible to pharmacologically modulate intrauterine adipogenesis by targeting trophoblast PPARγ.

### 2.4 Trophoblasts Increase the Differentiation of Preadipocytes via Intercellular Transfer of Exosomal PPARγ

In our experiments, coincubated 3T3‐L1 and HTR8 cells were separated by a membrane with 0.4‐µm pores. HTR8 cells might regulate biological processes in 3T3‐L1 cells by secreting free components and/or bioactive cargo‐containing vesicles with a small diameter, particularly exosomes.^[^ ^20^, ^21^ ^]^ Emerging evidence has shown a progressive increase in the abundance of placental exosomes in the maternal circulation throughout gestation.^[^ ^22^ ^]^ Disrupted placental exosomes are associated with complications such as FGR,^[^ ^23^ ^]^ suggesting a crucial role for exosomes in maternal–fetal communication. However, the occurrence of the transport of placental exosomes into the fetus and the involvement of these exosomes in the pathogenesis of FGR remain to be determined. We found that exosomes isolated from the culture medium of HTR8 cells (Figure S3A–C, Table S1, Supporting Information), but not exosome‐depleted culture medium, significantly increased oil red O staining in 3T3‐L1 cells; however, this effect was largely blunted for the exosomes derived from the PPARγ‐KD HTR8 cells (Figure 3C). This increase in adipogenesis was also observed in 3T3‐L1 cells coincubated with mouse trophoblast stem cells (mTSCs) or mTSC‐derived exosomes (mTSC‐Exos) (Figure S3D, Supporting Information). Moreover, exosomes isolated from PPARγ‐KD HTR8 cells failed to rescue lipogenesis in PPARγ‐KD 3T3‐L1 cells (Figure S3E, Supporting Information). Consistent with these findings, exosomes derived from the RGZ‐pretreated HTR8 cells significantly increased the differentiation of 3T3‐L1 cells, whereas additional pretreatment of HTR8 cells with GW9662 abolished this change (Figure 3D). Similarly, the recovery of PPARγ‐KD 3T3‐L1 cell differentiation by trophoblast‐derived exosomes was further promoted by activating PPARγ but abrogated by inhibiting PPARγ in HTR8 cells (Figure S3F, Supporting Information). In addition, the internalization of HTR8‐Exos by 3T3‐L1 cells was visualized (Figure S3G,H, Supporting Information). Consistent with these findings, the exosome secretion inhibitor GW4869^[^ ^24^ ^]^ significantly attenuated the stimulatory effects of HTR8 on 3T3‐L1 cell maturation without interfering with their differentiation when used alone (Figure 3E). Moreover, the uptake of HTR8‐Exos by 3T3‐L1 cells was substantially blocked in the presence of endocytosis inhibitors,^[^ ^25^ ^]^ and the differentiation of the cells was significantly limited (Figure S3I,J, Supporting Information).

### 2.5 Trophoblast PPARγ is the Dominant Contributor to the Effect of HTR8‐Exos on Preadipocyte Differentiation

Exosomes carry a broad spectrum of bioactive molecules, such as nucleic acids, mainly RNAs and proteins, that influence the activity and function of surrounding and distant cells.^[^ ^26^ ^]^ To identify the cargo in trophoblast‐derived exosomes that supports preadipocyte differentiation, we treated 3T3‐L1 cells with RNA and the RNA‐free content from HTR8‐Exos during differentiation. The RNA extracted from HTR8‐Exos did not retain the stimulatory effect of whole exosomes on 3T3‐L1 cell differentiation, whereas the RNA‐free content had a stimulatory effect (Figure 3F). Next, placental exosomes isolated from human pregnancies were subjected to proteomic analysis (Figure S4A–C, Table S1, Supporting Information), which confirmed the presence of PPARγ (Table S2, Supporting Information). In placental tissue, the abundance of p‐PPARγ^S112^ but not p‐PPARγ^S273^ was significantly increased, and the abundance of PPARγ was reduced in FGR‐affected placental exosomes (Figure 3G). A similar reduction in PPARγ abundance was also observed in the exosomes derived from PPARγ‐KD HTR8 cells (Figure S4D, Supporting Information). These results suggested that the PPARγ protein expressed in trophoblasts might be transported to fetal preadipocytes through exosomes to promote adipogenesis. However, whether insufficient exosome secretion from the placenta also contributes to compromised fetal adipogenesis remains unclear. We then determined that the number of placental exosomes was significantly greater in the fetuses with growth restriction than in the fetuses with normal growth (Figure S4E, Table S1, Supporting Information); this increase could represent adaptive compensation for the deficiency of lipogenesis in growth‐restricted fetuses. Conversely, ablation of PPARγ in HTR8 cells did not impact exosome secretion (Figure S4F, Table S1, Supporting Information).

### 2.6 Trophoblast‐Derived Exosomal PPARγ is Transported into the Nucleus of Preadipocytes during Differentiation

**Figure 4:**
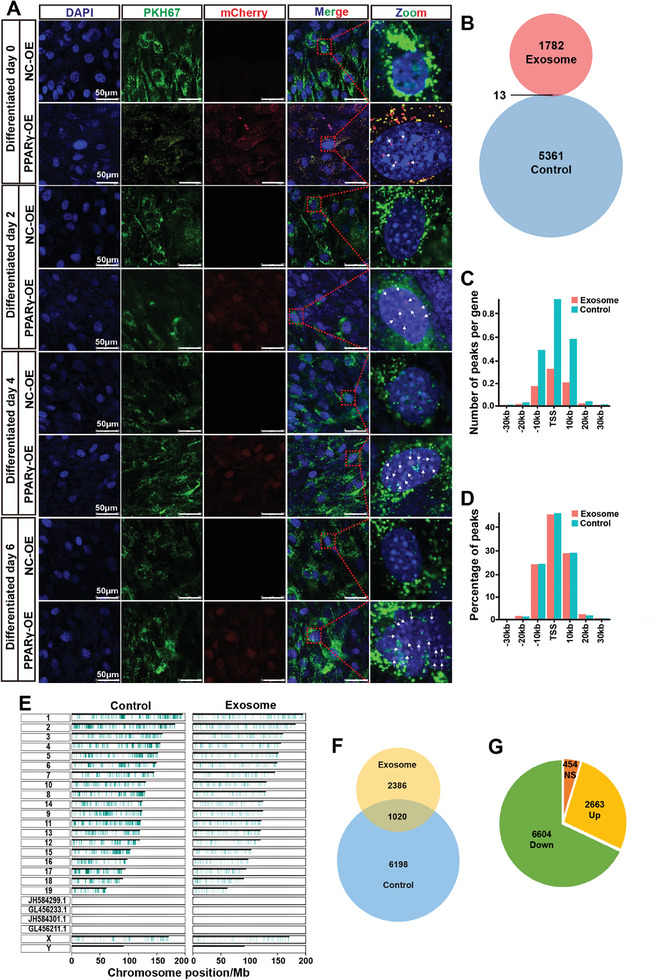
Trophoblast‐derived exosomal PPARγ directly regulates its target gene expression in preadipocytes. A) Confocal micrographs of mCherry‐fused exosomal PPARγ (red) in 3T3‐L1 cells after coculture with PKH67‐labeled exosomes (green) derived from PPARγ‐OE HTR8 cells on days 0, 2, 4, and 6 of differentiation. Scale bar: 50 µm. Arrow: mCherry‐fused exosomal PPARγ. *n* = 3 independent experiments with 3 independent preparations of exosomes. B) Venn diagram of ChIP‐seq peaks in the control group and exosomal PPARγ group. C,D) Distributions of peak numbers (C) and percentages (D) in the regions spanning ±30 kb with respect to the TSSs in the control and exosome groups. E) Gene modulator distribution on chromosomes in control and exosome‐treated 3T3‐L1 cells. F) Venn diagram of gene modulators that bind to mCherry‐PPARγ‐6His (exosome) and endogenous (control) PPARγ in 3T3‐L1 cells, as detected via ChIP‐seq. G) Pie chart of gene modulators with differential binding to exosomal mCherry‐PPARγ‐6His in 3T3‐L1 cells compared with endogenous PPARγ.

PPARγ is a member of the nuclear receptor superfamily of ligand‐inducible transcription factors and controls gene expression in gene networks involved in adipogenesis by binding to peroxisome proliferator‐responsive elements with RXR as obligate heterodimers.^[^ ^10^ ^]^ Therefore, the dominant, or “master”, transcription factor involved in the process of adipogenesis has long been recognized.^[^ ^7^, ^9^ ^]^ To determine whether the trophoblast PPARγ protein encapsulated in exosomes internalized into preadipocytes regulates the transcription of adipogenic genes, we generated an HTR8 cell line overexpressing the mCherry‐PPARγ‐6his fusion protein (PPARγ‐OE HTR8) and an HTR8 cell line expressing mCherry‐6his (NC‐OE HTR8) as the control (Figure S5A–C, Supporting Information). Interestingly, although comparable expression of mCherry‐ and His‐tagged proteins was observed in the above cells, mCherry‐ and His‐tagged proteins were barely detected in the exosomes derived from the NC‐OE HTR8 cells, in contrast to the exosomes derived from the PPARγ‐OE HTR8 cells (Figure S5D, Supporting Information). These findings indicate that trophoblasts contain the machinery for exosomal cargo sorting and loading.^[^ ^27^ ^]^ Next, exosomes derived from PPARγ‐OE HTR8 or NC‐OE HTR8 cells were applied to 3T3‐L1 cells on days −1, 1, 3, or 5 of differentiation, and the cells were incubated for 24 h. Exosome‐derived mCherry‐tagged PPARγ was visualized mainly in the cytoplasm of 3T3‐L1 cells on differentiation day 0, but the mCherry signal gradually overlapped with the DAPI signal on days 2, 4, and 6 (**Figure** **4**A). This phenomenon was not observed for exosome‐derived mCherry‐tagged Vimentin, which was expressed in the cytoplasm (Figure S5E–G, Supporting Information). This result suggested that the exosomal PPARγ protein released from HTR8 cells translocated into the nucleus of 3T3‐L1 preadipocytes, where it might function as an exogenous transcription factor.

### 2.7 Trophoblast‐Derived Exosomal PPARγ Regulates Transcription in Preadipocytes

**Figure 5:**
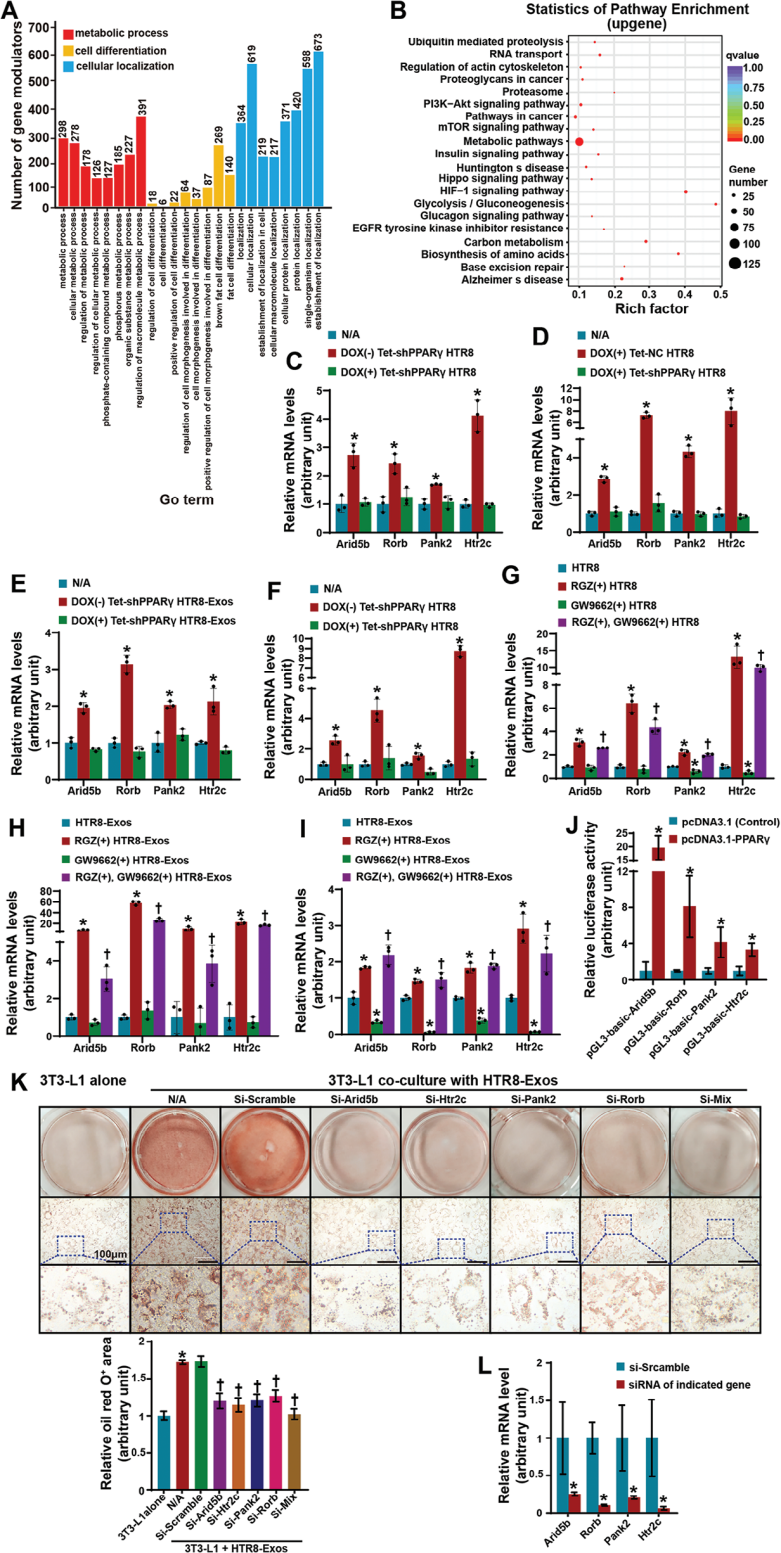
Trophoblast‐derived exosomal PPARγ interacts with adipogenic genes in preadipocytes. A,B) GO‐BP (A) and KEGG pathway (B) analyses of the upregulated gene modulators in the exosome group compared with those in the control group. C) qRT‒PCR analysis of *Arid5b, Rorb, Pank2*, and *Htr2c* in 3T3‐L1 cells on differentiation day 4 after coculture with untreated (N/A) or Tet‐shPPARγ HTR8 cells. *n* = 3 samples per group from 3 independent experiments. Two‐way ANOVA. *P < 0.05 versus N/A. D) qRT‒PCR analysis of *Arid5b, Rorb, Pank2*, and *Htr2c* in PPARγ‐KD 3T3‐L1 cells on differentiation day 4 alone (N/A) or cocultured with DOX‐treated Tet‐NC HTR8 cells or DOX‐untreated Tet‐shPPARγ HTR8 cells. *n* = 3 samples per group from 3 independent experiments. Two‐way ANOVA. *P < 0.05 versus N/A. E) qRT‒PCR analysis of *Arid5b, Rorb, Pank2*, and *Htr2c* in 3T3‐L1 cells on differentiation day 4 after coculture with no treatment (N/A) or exosomes from Tet‐shPPARγ HTR8 cells pretreated with DOX for 48 h or not treated with DOX. *n* = 3 samples per group from 3 independent experiments with independent preparations of exosomes. Two‐way ANOVA. *P < 0.05 versus N/A. F) qRT‒PCR analysis of *Arid5b, Rorb, Pank2*, and *Htr2c* in PPARγ‐KD 3T3‐L1 cells on differentiation day 4 after coculture with no treatment (N/A) or exosomes from Tet‐shPPARγ HTR8 cells pretreated with DOX for 48 h or not treated with DOX. *n* = 3 samples per group from 3 independent experiments with independent preparations of exosomes. Two‐way ANOVA. *P < 0.05 versus N/A. G) qRT‒PCR analysis of *Arid5b, Rorb, Pank2*, and *Htr2c* in PPARγ‐KD 3T3‐L1 cells on differentiation day 4 after coculture with HTR8 cells treated with RGZ, GW9662, or both. *n* = 3 samples per group from 3 independent experiments. Two‐way ANOVA. *P < 0.05 versus untreated cells. †*P *< 0.05 versus GW9662‐treated cells. H) qRT‒PCR analysis of *Arid5b, Rorb, Pank2*, and *Htr2c* in 3T3‐L1 cells on differentiation day 4 after coculture with exosomes derived from HTR8 cells pretreated with RGZ, GW9662, or both. *n* = 3 samples per group from 3 independent experiments with independent preparations of exosomes. Two‐way ANOVA. *P < 0.05 versus untreated HTR8‐Exos. †*P *< 0.05 versus GW9662‐treated HTR8‐Exos. I) qRT‒PCR of *Arid5b, Rorb, Pank2*, and *Htr2c* in PPARγ‐KD 3T3‐L1 cells on differentiation day 4 after coculture with exosomes derived from HTR8 cells pretreated with RGZ, GW9662, or both. *n* = 3 samples per group from 3 independent experiments with independent preparations of exosomes. Two‐way ANOVA. *P < 0.05 versus untreated HTR8‐Exos. #*P *< 0.05 versus GW9662‐treated HTR8‐Exos. J) Relative luciferase activity in HEK293 cells after cotransfection with the indicated vectors and PPARγ or control vectors. The data are expressed as fold changes relative to the luciferase activity measured in cells transfected with control vectors. *n *= 3 samples per group. One‐way ANOVA. *P < 0.05 versus the corresponding control. K) Oil red O‐stained 3T3‐L1 cells on differentiation day 8 after culture alone or coculture with HTR8‐Exos. The 3T3‐L1 cells in the coculture group were treated with N/A, Si‐scramble, Si‐*Arid5b*, Si‐*Htr2c*, Si‐*Pank2*, Si‐*Rorb*, or a mixture of Si‐*Arid5b*, Si‐*Htr2c*, Si‐*Pank2*, and Si‐*Rorb* (Si‐Mix) 48 h before the induction of differentiation and 2 h before the addition of HTR8‐Exos during differentiation. *n* = 3 independent experiments with 3 independent preparations of exosomes. Two‐way ANOVA. *P < 0.05 versus 3T3‐L1 cells cultured alone. †*P* < 0.05 versus N/A. Scale bar: 100 µm. L) qRT–PCR analysis of *Arid5b, Rorb, Pank2*, and *Htr2c* in 3T3‐L1 cells after treatment with *Arid5b, Rorb, Pank2*, or *Htr2c siRNA*. *n* = 3 samples per group. One‐way ANOVA. *P < 0.05 versus si‐scramble.

We next sought to verify the potential regulatory role of trophoblast‐derived exosomal PPARγ protein in gene expression in preadipocytes during differentiation. On differentiation day 3, 3T3‐L1 cells were treated with PPARγ‐OE HTR8‐Exos or HTR8‐Exos for 24 h. Then, Profinity IMAC resin and an antibody specific for PPARγ were used to pull down the PPARγ protein and bound DNA fragments in 3T3‐L1 cells. The chromatin immunoprecipitation (ChIP)‐seq results revealed 1795 and 5374 peaks in the exosomal PPARγ and endogenous PPARγ groups, respectively, with 13 overlapping peaks (Figure 4B). The peaks were unequally distributed on chromosomes 1–19 and the X chromosome and were highly enriched in the region within ±30 kb with respect to the transcription start site (TSS) (**Figure** **5**C–E), which is consistent with previously reported binding sites of PPARγ across species.^[^ ^28^ ^]^ Furthermore, 3406 and 7218 gene modulators were detected in the exosomal PPARγ and endogenous PPARγ groups, respectively, with 1020 overlapping gene modulators (Figure 4F). Differential expression analysis revealed 2663 upregulated and 6604 downregulated gene modulators in the exosomal PPARγ group compared with the endogenous PPARγ group, in addition to 454 nondifferentially expressed modulators (Figure 4G). Gene modulators of adipogenesis‐related factors, including *Arid5b*, *Agt*, *Htr2c*, *Rorb*, *Traf6*, *Tnfrsf1b*, *Mmp2*, *Gnas*, *Hexb*, *Ghr*, *Pank2*, *Serinc1*, *Runx1t1*, *Prkag3*, and *App*, exhibited the greatest enrichment in the Gene Ontology biological process (GO‐BP) and Kyoto Encyclopedia of Genes and Genomes (KEGG) pathway analyses (Figure 5A,B). Further validation experiments confirmed that the mRNA levels of *Arid5b*, *Rorb*, *Pank2*, and *Htr2c* in wild‐type and PPARγ‐KD 3T3‐L1 cells were significantly increased by coincubation with PPARγ‐expressing HTR8 cells or their derived exosomes on day 4 of differentiation (Figure 5C–F). However, such upregulation was not observed after PPARγ expression was ablated in HTR8 cells (Figure 5C–F). Moreover, the effects of incubation with RGZ‐ or GW9662‐pretreated HTR8 cells or their derived exosomes were consistent with the upregulation or downregulation of these genes in wild‐type and PPARγ‐KD 3T3‐L1 cells (Figure 5G–I). The results of luciferase reporter assays in HEK293 cells confirmed that PPARγ bound to the promoters of *Arid5b, Rorb, Pank2*, and *Htr2c* (Figure 5J). In support of these findings, the downregulated expression of *Arid5b, Rorb, Pank2*, and *Htr2c* in 3T3‐L1 cells strongly attenuated the increase in adipogenesis elicited by HTR8‐Exos (Figure 5K,L).

### 2.8 Mouse Placenta‐Derived Exosomes are Transported to the Fetus through the Umbilical Cord In Vivo

**Figure 6:**
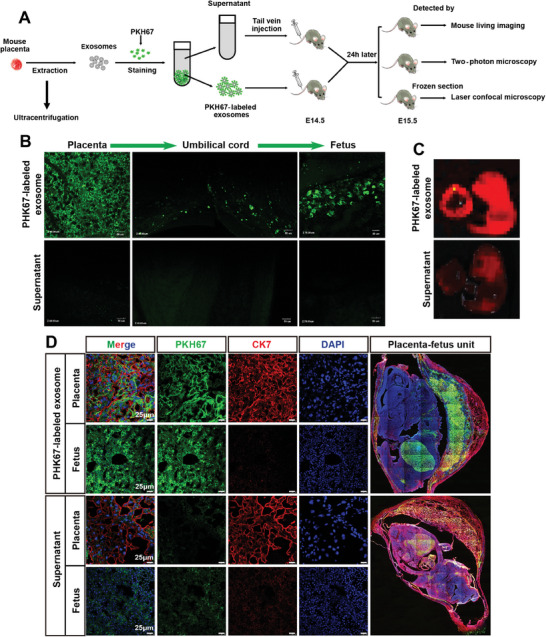
Mouse placental exosomes function as vehicles for the transport of cargos to the fetus through the fetal–placental blood circulation. A) Schematic showing the experimental design for tracing placenta‐derived exosomes in pregnant mice. Exosomes isolated from 50 mg of mouse placental tissue were labeled with PKH67. Pregnant mice (GD 14.5) were subsequently injected with PKH67‐labeled exosomes or supernatants (the last wash for PKH67 exosome labeling), and PKH67 signals were detected 24 h later. B) Two‐photon micrographs of PKH67‐labeled exosomes (green) in the placenta, umbilical cord, and fetus of living mice. C) Images of PKH67 fluorescence (red) in the dissected placenta and fetus acquired with an IVIS Spectrum optical imaging system. *n* = 13 litters from 3 dams in each group; one representative image from each group is shown. D) Confocal micrographs of PKH67‐labeled exosomes (green), CK7 staining (red), and DAPI staining (blue) in frozen sections of placental–fetal units. Scale bar: 25 µm. *n* = 14 litters from 3 dams in each group; one representative image from each group is shown.

To trace the trafficking of placenta‐derived exosomes to fetuses in vivo, we identified exosomes isolated from mouse placentas and stained them with PKH67 (Figure S6A–C, Table S1, Supporting Information) prior to transplantation into pregnant mice on gestational day (GD) 14.5 via tail vein injection (**Figure** **6**A). Twenty‐four hours later, two‐photon microscopy revealed a substantial accumulation of PKH67‐stained exosomes in the placental labyrinth, vessel lumens in the umbilical cord, and fetal abdominal tissues of living pregnant mice (Figure 6B, Video S1, Supporting Information). Next, the dissected fetuses and placentas were examined by ex vivo imaging, and the PKH67 signal was visible in both the placenta and the fetus (Figure 6C). Furthermore, the PKH67 signal was detected in frozen sections of whole placental–fetal units. Our data clearly revealed that placental exosomes were enriched not only in the liver but also in subcutaneous fetal tissue (Figure 6D), thus explaining the detection of the PKH67 signal throughout the fetus following transplantation of placenta‐derived exosomes via ex vivo imaging. Given that the umbilical vein supplies blood from the placenta directly to the fetal liver through the portal vein and ductus venosus, these findings clearly delineate the delivery path of placenta‐derived exosomes to the fetus and highlight a critical role for the placenta in the regulation of fetal fat development in utero.

### 2.9 Placenta‐Specific Activation of PPARγ in Mice Achieved by Nanoparticle‐Delivered RGZ

Our in vitro experimental data revealed that the activation of placental trophoblast PPARγ by RGZ was beneficial for exosome‐mediated preadipocyte differentiation, yet the clinical application of RGZ during pregnancy is contraindicated, not only because of associated cardiovascular events^[^ ^29^ ^]^ but also because of the potential risk for major birth defects, stillbirth, and macrosomia‐related morbidity, as indicated by limited evidence from animal studies. To minimize potential adverse effects from the systemic use of RGZ during gestation, we developed a method for placenta‐targeted RGZ administration using novel nanoparticles conjugated to placental chondroitin sulfate A (CSA)‐binding peptide (plCSA‐NPs).^[^ ^30^ ^]^ Indocyanine green (ICG)‐loaded nanoparticles (INPs) and ICG‐loaded plCSA‐NPs (plCSA‐INPs) were administered to pregnant mice on GD 14.5 via intravenous injection. Live animal imaging confirmed that plCSA‐INP administration resulted in substantially stronger ICG fluorescence in the placenta than did INP administration. Most importantly, the ICG signal was exclusive to the fetus (Figure S6D, Supporting Information). Compared with RGZ dissolved in dimethyl sulfoxide (DMSO), RGZ‐loaded plCSA‐unconjugated nanoparticles (RNPs) and placenta‐targeted NPs (plCSA‐RNPs) resulted in significantly longer retention of RGZ in the circulation of pregnant mice following intravenous injection (Figure S6E, Supporting Information), confirming the slow release of RGZ by the NPs. Moreover, although circulatory RGZ was barely detected in dams after the administration of 0.1 mg kg^−1^ plCSA‐RNPs, this dose sufficiently suppressed the phosphorylation of PPARγ^S112^ in placental tissues for at least 24 h without disrupting PPARγ phosphorylation in the heart, liver, or uterus (Figure S6E,F, Supporting Information).

### 2.10 Placenta‐Specific Activation of PPARγ Rescues FGR by Normalizing Fetal Adipogenesis

**Figure 7:**
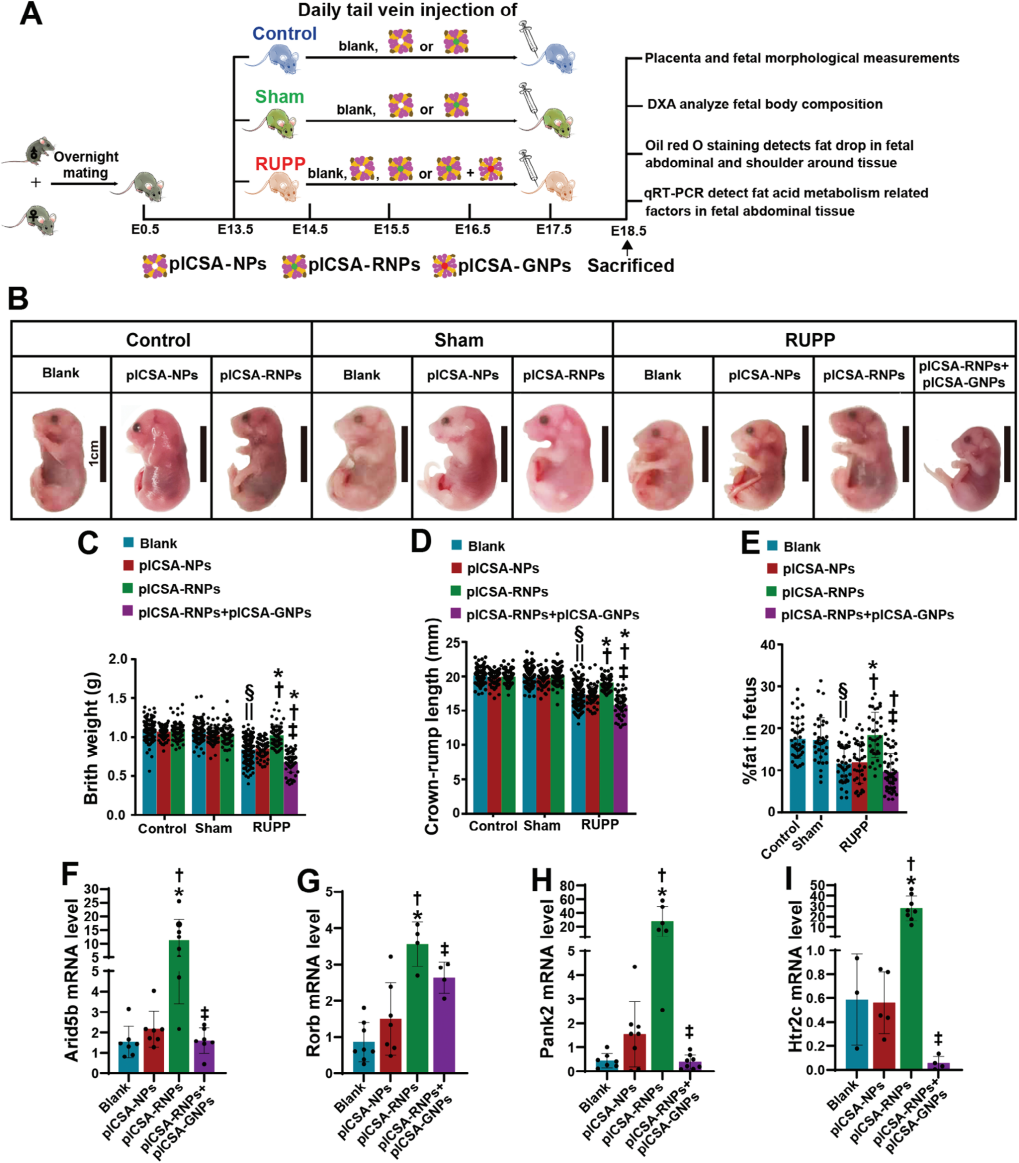
Placenta‐specific activation of PPARγ rescues FGR by improving adipogenesis through placenta‐derived exosomes. A) Diagram of the experimental design. B–D) Representative images of fetuses (B) and analysis of fetal BW (C) and CRL (D) in fetuses from dams in the control, sham, and RUPP groups subjected to the indicated treatments. Scale bar: 1 cm. Control‐Blank, *n* = 150 litters from 22 dams. Control‐plCSA‐NPs, *n* = 92 litters from 12 dams. Control‐plCSA‐RNPs, *n* = 88 litters from 12 dams. Sham‐Blank, *n* = 125 litters from 20 dams. Sham‐plCSA‐NPs, *n* = 85 litters from 12 dams. Sham‐plCSA‐RNPs, *n* = 77 litters from 12 dams. RUPP‐Blank, *n* = 164 litters from 30 dams. RUPP‐plCSA‐NPs, *n *= 69 litters from 12 dams. RUPP‐plCSA‐RNPs, *n* = 57 litters from 12 dams. RUPP‐plCSA‐RNPs plus plCSA‐GNPs, *n* = 58 litters from 12 dams. Two‐way ANOVA. *P < 0.05 versus RUPP‐Blank. †*P *< 0.05 versus RUPP‐plCSA‐NPs. ‡*P *< 0.05 versus RUPP‐plCSA‐RNPs. §*P *< 0.05 versus Control‐Blank. ||*P *< 0.05 versus Sham‐Blank. E) Fetal body fat composition, as assessed by DEXA scanning. Control‐Blank, *n* = 41 litters from 8 dams. Sham‐Blank, *n *= 30 litters from 7 dams. RUPP‐Blank, *n* = 39 litters from 6 dams. RUPP‐plCSA‐NPs, *n* = 35 litters from 6 dams. RUPP‐plCSA‐RNPs, *n* = 35 litters from 8 dams. RUPP‐plCSA‐RNPs plus plCSA‐GNPs, *n* = 49 litters from 7 dams. Two‐way ANOVA. *P < 0.05 versus RUPP‐Blank. †*P *< 0.05 versus RUPP‐plCSA‐NPs. ‡*P *< 0.05 versus RUPP‐plCSA‐RNPs. §*P *< 0.05 versus Control‐Blank. ||*P *< 0.05 versus Sham‐Blank. F) qRT–PCR of *Arid5b, Rorb, Pank2*, and *Htr2c* in the ASF of fetuses in the RUPP group subjected to the indicated treatments. Two‐way ANOVA. *P < 0.05 versus RUPP‐Blank. †*P *< 0.05 versus RUPP‐plCSA‐NPs. ‡*P *< 0.05 versus RUPP‐plCSA‐RNPs. For cotreatment with plCSA‐RNPs plus plCSA‐GNPs, plCSA‐GNPs were administered 2 h prior to plCSA‐RNPs.

To evaluate the therapeutic potential of targeting placental PPARγ to treat FGR, we generated low‐protein diet (LPD)‐induced and reduced uterine perfusion pressure (RUPP)‐induced FGR models in mice (Figure S7A–E, Supporting Information). The hyperphosphorylation of PPARγ^S112^ in both placental tissues and placenta‐derived exosomes was reproduced in the RUPP model but not in the LPD model (Figure S7F–H, Supporting Information), with no disruption of exosome secretion observed (Figure S7I, Table S1, Supporting Information). The dams in the RUPP, sham, and control (no surgery) groups were then treated daily with 0.1 mg kg^−1^ plCSA‐RNPs or 0.1 mg kg^−1^ plCSA‐NPs via tail vein injection from GD 13.5 to 17.5 or left untreated (blank group) (**Figure** **7**A). Strikingly, the significant reductions in BW and crown–rump length (CRL) of the offspring in the RUPP group were reversed by the injection of plCSA‐RNPs but not plCSA‐NPs, whereas none of the offspring in the control and sham groups presented alterations in fetal BW or CRL (Figure 7B–D). These results indicated that preventative use was unlikely to increase the risk of macrosomia. Most importantly, dual‐energy X‐ray absorptiometry (DEXA) revealed that the compromised body fat composition of the fetuses in the RUPP group was substantially ameliorated by treatment with plCSA‐RNPs (Figure 7E). Specifically, adipogenesis in shoulder‐adjacent and abdominal tissues, which was notably suppressed in the fetuses in the RUPP group, was strongly increased by treatment with plCSA‐RNPs (Figure S7J, Supporting Information). Importantly, specific inhibition of exosome secretion in the placenta by the administration of GW4869‐loaded plCSA‐NPs (plCSA‐GNPs) abrogated the benefits of plCSA‐RNPs in fetuses in the RUPP group (Figure 7B–E) without compromising placental development (Figure S7K, Supporting Information). Furthermore, *Arid5b*, *Rorb*, *Pank2*, and *Htr2c* were significantly upregulated in fetal ASF tissue in the RUPP group by plCSA‐RNP treatment; however, this upregulation was abolished by the additional administration of plCSA‐GNPs (Figure 7F).

RGZ is beneficial for lowering blood pressure (BP) by modulating the renin‒angiotensin II system.^[^ ^31^ ^]^ Notably, neither plCSA‐NPs nor plCSA‐RNPs ameliorated RUPP‐induced gestational hypertension, elevation of serum soluble FMS‐like tyrosine kinase‐1 (sFlt1), or morphological changes in the kidney (Figure S7L–N, Supporting Information). These findings strongly suggested that the rescue of the FGR phenotype in the RUPP model through PPARγ agonist treatment was independent of any effects on gestational hypertension.

### 2.11 Placenta‐Specific RGZ Administration Could be a Safe Therapy for Treating FGR

RGZ is a potent insulin sensitizer and is thus used to lower blood glucose in humans with type 2 diabetes. However, hypoglycemia during gestation substantially limits the supply of glucose, the fundamental energy source for the fetus, and this phenomenon may be related to the fetal death observed with oral RGZ treatment during pregnancy.^[^ ^32^ ^]^ In contrast, in the present study, placenta‐targeted administration of RGZ via plCSA‐NPs did not affect the maternal blood glucose level (Figure S8A, Supporting Information), likely because of not only the low dose of RGZ but also its slow release and targeted delivery via plCSA‐NPs.

However, as shown in Figure 6B–D, placental exosomes were transported to the fetus through the umbilical vein and accumulated in the fetal liver. Given that placental exosomes carry substantial amounts of PPARγ to stimulate adipogenesis, we evaluated the potential of plCSA‐RNPs to increase the risk of fatty liver in the fetus. Our data revealed that targeted activation of placental PPARγ by plCSA‐RNP treatment did not alter the size or morphology of fetal liver cells, and structural observation of the fetal liver and placenta revealed no differences among the control, sham, and RUPP groups (Figure S8B,C, Supporting Information). Consistent with these findings, the triglyceride (TG) and cholesterol levels in the fetal liver were not disrupted by plCSA‐RNP treatment (Figure S8D,E, Supporting Information).

Bone loss is a well‐documented side effect of RGZ.^[^ ^10^ ^]^ Since both osteoblasts and adipocytes are derived from a common mesenchymal progenitor cell (MSC) and since there is an inverse relationship between the gene expression dynamics of these two lineages,^[^ ^33^ ^]^ we investigated whether increased adipogenesis can compromise fetal osteogenesis and skeletal development. We found that the bone mineral density did not differ among fetuses in the RUPP group (Figure S8F, Supporting Information). Moreover, plCSA‐RNP treatment did not affect the proportion of mature bone or cartilage in the fetus (Figure S8G–K, Supporting Information). Although the transport of exosomal PPARγ originating from HTR8 cells into the nuclei of 3T3‐L1 cells was detected on day 0 of differentiation induction, the number of exosomal PPARγ molecules originating from HTR8 cells that entered the nucleus in 3T3‐L1 cells increased substantially on day 4 of differentiation induction, indicating that PPARγ resulted in normalization of only the mid‒late stage of differentiation from committed preadipocytes to mature adipocytes and was therefore unlikely to influence cell fate determination in MSCs.

## 3 Discussion

The etiology of FGR is poorly understood, and no effective pharmacological intervention except for termination of pregnancy is available for this condition. Intriguingly, our data revealed that although PPARγ expression is much higher in the placenta than in fetal ASF tissue throughout gestation, few lipid droplets were detected in the placenta, indicating that the placenta might be a reservoir of PPARγ for the fetus during late gestation. Consistent with these hypotheses, we also observed high PPARγ expression in both human trophoblasts and murine placental tissues at different gestational ages. Although multiple transcription factors play a role in adipogenesis, we found that neither C/EBPα nor SREBP1 is the primary driver of terminal differentiation of preadipocytes. The expression of PPARγ with a nonphosphorylatable mutation at S112 yielded cells with increased sensitivity to ligand‐induced adipogenesis,^[^ ^16^ ^]^ whereas FGR‐affected placentas presented an increased level of p‐PPARγ^S112^, indicating a substantial decrease in PPARγ‐dependent adipogenesis.

The functions of PPARγ are regulated by various post‐translational modifications, especially phosphorylation. Numerous reports have shown that PPARγ is phosphorylated upon activation of the MAPK pathway in various cells, including trophoblasts.^[^ ^34^, ^35^ ^]^ The phosphorylation of PPARγ at both Ser112 and Ser273 can be blocked by thiazolidinedione (TZD) full PPARγ agonists, such as RGZ, and this blockade is followed by increased transcriptional activity of genes related to adipogenesis and insulin sensitivity.^[^ ^36^, ^37^ ^]^ Barak and colleagues demonstrated that PPARγ mutations lead to embryonic lethality and deficiency in all forms of fat in mice.^[^ ^12^ ^]^ However, PPARγ‐null embryos can be rescued by tetraploid complementation or by preserving PPARγ expression in the trophoblast,^[^ ^12^, ^13^ ^]^ suggesting that the placenta plays a decisive role in the regulation of intrauterine fat development by supplying PPARγ to the fetus. Similarly, human placental PPARγ expression was reported to be positively associated with placental and fetal weights at birth, particularly within the small for gestational age subpopulation^[^ ^38^ ^]^ Nevertheless, accumulating studies have shown that PPARγ plays important regulatory roles not only in trophoblast stem cell differentiation^[^ ^11^ ^]^ but also in human chorionic gonadotropin hormone (HCG) secretion^[^ ^39^ ^]^ and nutrient transportation from the placenta to the fetus^[^ ^32^, ^40^, ^41^ ^]^ These findings indicate that PPARγ is critical for placental development and function; therefore, the promoting effects of placental PPARγ on fetal fat development have long been assumed to be secondary to improved placental development and function.

Here, via studies in *PPARγ^flox/flox^; ADACre^+/+^* mice, ablation of placental PPARγ was induced beginning on GD13.5, after the placenta had fully developed. Moreover, to avoid the potential toxicity of DOX on the placenta and fetal growth in mice,^[^ ^42^, ^43^ ^]^ we selected a lower dosage of DOX (0.5 mg ml^−1^) in this study to induce *Cre* expression, which did not disturb placental or fetal weight at birth in the control mice. Our results clearly demonstrated for the first time that insufficient PPARγ expression in a normally developing placenta only during late gestation effectively causes defective adipogenesis in the fetus, a major manifestation of FGR.

Among various PPARγ‐expressing cells derived from the fetal–maternal interface, only trophoblasts exhibit significantly increased differentiation into preadipocytes under noncontact coculture conditions, which is consistent with evidence indicating that the placenta releases exosomes to modulate fetal–placental and/or fetal–maternal communication to support pregnancy.^[^ ^44^ ^]^ These findings strongly support the critical role of the placenta in the regulation of fat development in utero, which is dependent on PPARγ expression in trophoblasts. Moreover, we determined that microtubule‐, clathrin‐, and caveolin‐dependent internalization contributed to the uptake of trophoblast‐derived exosomes by preadipocytes, suggesting that exosome‐mediated substance transport may be a widespread major mode of intercellular communication for preadipocytes and is therefore involved in their differentiation. Most importantly, through the tracing of PKH67‐labeled trophoblast‐derived exosomes in vivo after transplantation into pregnant mice, the present study provides the first direct evidence of the transport of exosomes from placental trophoblasts to the fetus, indicating that the placenta might play a much more profound but undefined role in the regulation of fetal development.

The results of expression profiling studies during adipocyte differentiation and subsequent PPARγ ligand treatment suggest that hundreds of genes may be regulated by PPARγ^[^ ^45^ ^]^ and that binding sites for PPARγ are near most genes induced during adipogenesis,^[^ ^46^ ^]^ indicating that PPARγ is directly involved in the activation of most adipocyte‐specific genes. The most important and specific factor in adipogenic differentiation, PPARγ, is transcriptionally induced during the 2 days after induction of differentiation, and its expression peaks by days 3–4.^[^ ^17^, ^47^ ^]^ To investigate the differences in the regulation of downstream genes between mouse endogenous PPARγ and exogenous PPARγ in trophoblasts, we subjected sheared DNA decrosslinked from an anti‐PPARγ antibody in 3T3‐L1 cells and from Profinity IMAC resin in HTR8‐Exo‐treated 3T3‐L1 cells on differentiation day 4 to ChIP‐seq to determine the binding sites of PPARγ from various sources in 3T3‐L1 cells. Genes that bind to endogenous PPARγ, such as *FABP4*, *CD36*, *Lipe*, *Olr1*, and *Me1*, were previously discovered in other studies.^[^ ^17^ ^]^ However, analysis of trophoblast DNA bound to exosomal PPARγ in preadipocytes revealed a different spectrum of genes closely related to cell differentiation and adipogenesis, including *Arid5b, Rorb, Pank2*, and *Htr2c*. Thus, we identified a novel mechanism and downstream signaling pathway involved in trophoblast‐stimulated preadipocyte differentiation.

In this study, we reported compromised activity of PPARγ in exosomes derived from human FGR‐affected placentas and mouse placentas in the RUPP model. We also demonstrated that exosome‐dependent proadipogenic effects could be regulated by modulating PPARγ expression in HTR8 trophoblasts. For evaluation of the therapeutic potential of RGZ in targeting placental PPARγ for FGR management, RGZ was administered to RUPP model mice via plCSA‐NPs conjugated to VAR2CSA, a CSA‐binding protein that binds specifically to trophoblasts in the placenta.^[^ ^30^ ^]^ Owing to the placenta‐targeting and slow‐release properties of plCSA‐NPs, 0.1 mg kg^−1 ^day^−1^ RGZ effectively increased PPARγ activity in the placenta, which was nearly undetectable in the maternal peripheral blood 4 h after treatment. This dosage is far lower than the dosages of RGZ known to ameliorate both hypertension and endothelial dysfunction.^[^ ^48^ ^]^ Furthermore, no interference with fetal or placental development has been observed in rodents,^[^ ^32^, ^49^ ^]^ and no unexpected PPARγ activation in major maternal organs or adverse effects in dams has been reported.

Our data demonstrate that plCSA‐RNPs increase fetal adipogenesis and growth in FGR‐affected mouse pups. PPARγ activation in human trophoblasts increases the uptake of free fatty acids, upregulates the expression of fatty acid transport‐related proteins, and increases the transport of fatty acids from the placenta to the fetus.^[^ ^32^, ^40^ ^]^ However, the effects of plCSA‐RNPs on fetal growth are abolished by the application of an additional exosome secretion inhibitor in the placenta, indicating that the influence of trophoblast PPARγ on fetal adipogenesis depends on exosomes. Moreover, potential adverse effects of RGZ, such as cardiovascular accidents, hypoglycemia, and skeletal maldevelopment, were prevented by the use of plCSA‐RNPs in our study. Moreover, a detailed neonatal examination did not reveal any evidence of external, cardiac, pulmonary, or gastrointestinal congenital malformations (data not shown). These findings provide a new method of RGZ administration and indicate that the classic hypoglycemic drug RGZ can play a positive role in the treatment of FGR. The off‐label use of this drug contributes to the innovativeness of this study.

Nevertheless, we should be aware of the complexity of exosomal cargos. In addition to proteins, exosomes derived from trophoblasts present a profile of growth factors, DNA fragments, mRNAs, miRNAs, and phospholipids.^[^ ^50^ ^]^ Given that the content, biogenesis, and release of exosomes are intricately tied to the microenvironment of the placenta^[^ ^50^, ^51^ ^]^ various exosomal cargoes other than PPARγ may also be involved in the regulation of fetal adipogenesis. Moreover, exosomes participate in the regulation of the maternal immunologic response, cellular metabolic homeostasis, fetal vasculogenesis, maternal uterine vascular tone, and metabolic adaptation during gestation,^[^ ^52^ ^]^ all of which may contribute to the modulation of in utero fat development in concert with the adipogenic gene expression driven by PPARγ.

The lack of conclusive in vivo and in vitro models is a recognized challenge for studies on human placental development and function. In the present work, immortalized HTR8/SVneo human trophoblast cells were used for most in vitro experiments because we revealed that HTR8/SVneo cells not only consistently express high levels of PPARγ but also replicate the exosomal PPARγ abundance and exosome release features of human term placenta‐derived primary trophoblasts (Figure S9, Supporting Information). Although validating PPARγ deficiency in primary trophoblasts is infeasible because of rapid spontaneous syncytialization, primary trophoblasts significantly improved 3T3‐L1 cell differentiation in coculture experiments, confirming the findings in HTR8/SVneo cells.

In summary, this study reveals a novel mechanism for in utero fat development that is dependent on trophoblast‐derived exosomes and PPARγ. Our work highlights the placenta not only as a unique venue for fetal–maternal material exchange but also as an important regulatory factor in fetal growth and development, despite its ephemeral status. The complexities of placental function may far exceed our current understanding and thus merit more attention and research.

## 4 Experimental Section

### Clinical Characteristics

Women with FGR‐complicated pregnancies (*n* = 35) and women with normal pregnancies (*n *= 35) were recruited from the Department of Obstetrics, the First Affiliated Hospital of Chongqing Medical University (Table S3, Supporting Information). Informed consent was obtained from each participant. The inclusion criteria for women with normal pregnancies (normal group) were as follows: singleton pregnancy, no pregnancy complications or coexisting disease, and normal fetal growth and fetal BW between the tenth and 90th percentiles for the same gestational age. The inclusion criteria for women with pregnancies complicated with FGR (FGR group) were as follows: failure of the fetus to reach its expected biological potential in terms of physique, singleton pregnancy, no other pregnancy complications or coexisting disease, and a fetal BW less than the fifth percentile for the same gestational age.^[^ ^53^ ^]^ In addition, women with normal pregnancies that were terminated before 37 weeks of gestation for personal reasons were recruited from the Department of Obstetrics and Gynecology, Chongqing Yubei District Maternal and Child Health Care Hospital (Table S4, Supporting Information). Early‐ and midgestation placenta and fetal ASF tissue samples were collected from women with normal pregnancies who voluntarily chose legal termination for nonmedical reasons. All the sample collection procedures referred to in this experiment were completed with the informed consent of the pregnant women and in accordance with the National Institutes of Health guidelines, and they were approved by the Institutional Ethics Committee of Chongqing Medical University. The ethical approval numbers for this study were 2018‐011 and 2020–788. The first day of the last menstrual period was defined as the first day of gestation.

### Sample Collection

Human sample sampling was performed as previously described, with modifications.^[^ ^54^ ^]^ In brief, the chorionic villus, the maternal surface of the placenta, and the ASF were randomly cut with scissors and were washed thoroughly but gently in cold physiological saline. For protein and exosome extraction, the samples were divided into 50 mm^3^ (50 mg) pieces and then stored at −80 °C for subsequent use after being snap frozen in liquid nitrogen. For RNA extraction, the samples were immersed in 1 ml of RNAlater (QIAGEN, Germany) after being divided into 5 mm^3^ (5 mg) pieces and then stored at −80 °C. For immunofluorescence and oil red O staining, the samples were divided into 0.5 cm^3^ (pea‐sized) pieces, dehydrated in 5–10 ml of 25% sucrose overnight after fixation with 4% paraformaldehyde (PFA) at 4 °C for 1 h, sliced into 10‐µm sections via a freezing microtome after immersion in O.C.T. compound (Sakura, USA), and then stored at −80 °C. All the sample collection procedures referred to in this experiment were completed with the informed consent of the pregnant women and in accordance with the National Institutes of Health guidelines, and they were approved by the Institutional Ethics Committee of Chongqing Medical University.

### Isolation of Human Term Placenta‐Derived Primary Trophoblasts

The methods used for the extraction and validation of human term placenta‐derived primary trophoblasts were consistent with those previously published by our research group.^[^ ^55^ ^]^ The primary trophoblasts in the present study were isolated from healthy human term placentas and cultured in Dulbecco's modified Eagle's medium (DMEM/F12; Gibco, USA) supplemented with 5 µg ml^−1^ insulin (Sigma–Aldrich, Germany), 10 µg ml^−1^ transferrin (Sigma–Aldrich, Germany), 20 nm sodium selenite (Sigma–Aldrich, Germany), 5 ng ml^−1^ EGF (Sigma–Aldrich, Germany), 400  U l^−1^ hCG (Abcam, UK), 10% FBS 2 (22 XU ET AL) (Gibco, USA), 1% penicillin/streptomycin (PS, Gibco, USA), 50 µg ml^−1^ gentamycin (Gibco, USA) and 0.25 µg ml^−1^ amphotericin (Gibco, USA). The cell medium was replaced with the same medium prepared with exosome‐depleted FBS obtained by ultracentrifugation for 16 h at 160 000 × g and 4 °C prior to filtration through a 0.22‐µm filter before exosome‐related experiments were conducted.

### Cell Culture

The immortalized trophoblast line HTR8/SVneo (HTR8) was purchased from the American Type Culture Collection (ATCC; USA). The 3T3‐L1 preadipocyte line was a kind gift from Professor Fiona M. Watt (King's College London). The human umbilical vein endothelial cell (HUVEC) line, uterine smooth muscle HUM cell line, amniotic epithelial WISH cell line, and the human choriocarcinoma cell lines JEG3 and BeWo were purchased from the Chinese Academy of Sciences. All the cell lines were assessed monthly for mycoplasma contamination. HTR8 cells and HUVECs were routinely cultured in basal RPMI‐1640 medium (Gibco, USA) supplemented with 10% fetal bovine serum (FBS; Gibco, USA) and 1% PS (Gibco, USA). 3T3‐L1 cells were routinely cultured in a growth medium consisting of high‐glucose DMEM (Gibco, USA), 10% FBS (Gibco, USA), and 1% PS. WISH cells were routinely cultured in a growth medium consisting of Opti‐Minimal Essential Medium (Opti‐MEM; Gibco, USA), 10% FBS, and 1% PS. HUM cells were routinely cultured in a growth medium consisting of high‐glucose DMEM, 10% FBS, and 1% PS. JEG3 cells were routinely cultured in DMEM/F12 (Gibco, USA) supplemented with 10% FBS and 1% PS. BEWO cells were routinely cultured in DMEM (Gibco, USA), 10% FBS, and 1% PS. All the cells were cultured at 37 °C in a humidified atmosphere with 5% CO_2_. The culture medium was changed every other day, and the cells were passaged after they reached 70% to 80% confluence.^[^ ^56^ ^]^ All the cell culture medium was replaced with the same medium prepared with exosome‐depleted FBS obtained by ultracentrifugation for 16 h at 160 000 × g and 4 °C prior to filtration through a 0.22‐µm filter before exosome‐related experiments were conducted.

### Generation of Mouse Embryonic Fibroblasts (MEFs) and Fibroblast‐Conditioned Medium (FCM)

MEFs were derived as previously described.^[^ ^57^ ^]^ After filtration, centrifugation, and washing, GD 13.5 fetuses whose heads, limbs, tails, and most of the internal organs were removed were minced, trypsinized for 10 min, and then seeded into 100‐mm cell culture dishes in 10 ml of DMEM supplemented with 10% FBS and 1% PS. The cells were split at a 1:2 ratio when they reached confluence and were then treated with 10 µg ml^−1^ mitomycin C (MMC) for 2 h after two or three passages. The cells were subsequently cultured in trophoblast stem cell medium (TSM) (RPMI 1640 medium supplemented with 20% FBS, 1% PS, 1 mm sodium pyruvate, 100 µm β‐mercaptoethanol and 2 mm *L*‐glutamine), and the supernatant was collected three times at three‐day intervals and called the FCM.

### mTSC Culture

Mouse TSCs were derived from the trophectoderm as previously described.^[^ ^58^ ^]^ In brief, blastocysts obtained from the uterus on GD 3.5 were seeded onto MMC‐treated MEFs and cultured in TSM + F4H (25 ng ml^−1^ FGF4 and 1 µg ml^−1^ heparin) until outgrowth formation. Several days after the outgrowth disaggregated, mTSC colonies arose and were passaged 7–10 times; the passaged cells were also plated onto MMC‐treated MEFs and cultured in TSM + F4H. Finally, the mTSCs were cultured in 70% FCM + F4H (containing 70% FCM and 30% TSM) when they exhibited stable growth.

### 3T3‐L1 Cell Differentiation

The procedure for 3T3‐L1 preadipocyte differentiation was carried out as described in previous studies, with modifications.^[^ ^59^ ^]^ In brief, when 3T3‐L1 cells were 100% confluent, differentiation was induced with differentiation solution A [DMEM high glucose supplemented with 10% FBS, 1% PS, 1 µg ml^−1^ insulin (Merck, Germany), 1 µm dexamethasone (Sigma–Aldrich, Germany), and 0.5 mm 3‐isobutylmethylxanthine (IBMX; Sigma–Aldrich, Germany)]. Two days later, differentiation solution A was replaced with differentiation solution B (high‐glucose DMEM supplemented with 10% FBS, 1% PS, and 1 µg ml^−1^ insulin), and the culture was continued for another two days. The differentiated cells were then maintained in differentiation solution C (high‐glucose DMEM supplemented with 10% FBS and 1% PS), and the medium was changed every other day. On the eighth day of differentiation, differentiated cells were subjected to oil red O (Sigma–Aldrich, Germany) staining.

### Construction and Transduction of the PPARγ‐Knockdown Lentiviral Vector Controlled by the Tet‐on System (Tet‐shPPARγ)

Lentiviral vectors were produced as previously described.^[^ ^60^ ^]^ In brief, lentiviruses were generated via the transfection of the lentiviral plasmid TetIIP‐TurboRFP‐MCS (MIR30)‐Ubi‐TetR‐IRES‐puromycin into HTR8 and 3T3‐L1 cells in the presence of transfection reagent (GENE, Shanghai, China). The PPARγ knockdown sequence (5'‐CAGCATTTCTACTCCACAT‐3') or scramble control sequence (5'‐TCTCGCTTGGGCGAGAGTAAG‐3') was inserted into the MCS after digestion with the restriction enzymes XhoI and EcoRI. The lentivirally transduced cells were screened via incubation with 2 µg ml^−1^ puromycin, and expression was induced with DOX (Selleck, USA). The Tet‐shPPARγ efficacy was determined via quantitative real‐time PCR (qRT–PCR) or western blotting after treatment with DOX for 24 or 48 h, respectively.

### Construction and Transduction of Lentiviral Vectors for PPARγ and Vimentin Overexpression

The retroviral plasmid vector GV348 for PPARγ or Vimentin overexpression (PPARγ‐OE or Vimentin‐OE) consisting of the fused recombinant mCherry sequence, full‐length PPARγ or Vimentin sequence (Gene ID: NM_011146 for PPARγ, NM_011701 for Vimentin) and recombinant 6His sequence, and the negative control overexpression (NC‐OE) retroviral vector GV348 containing the fused recombinant mCherry and 6His sequences, were purchased from GENE (Shanghai, China). The element sequence of GV348 was Ubi‐MCS‐SV40‐puromycin, and the fusion sequence was inserted into the MCS after digestion with the restriction enzymes AgeI and EcoRI. HTR8 cells were infected with lentiviruses containing the PPARγ‐OE, Vimentin‐OE, or NC‐OE retroviral vector in the presence of a transfection reagent (GENE, Shanghai, China). The lentivirally transduced HTR8 cells were purified via selection with 2 µg ml^−1^ puromycin, and the PPARγ‐OE efficiency was evaluated by measuring the mRNA and protein levels via qRT–PCR and western blotting, respectively.

### In Vitro Pharmacokinetic Analysis of RGZ and GW9662

RGZ (Sigma–Aldrich, USA) and GW9662 (Sigma–Aldrich, USA) were dissolved in DMSO (Sigma–Aldrich, Germany) to a final DMSO concentration of less than 0.1% in the medium. The optimal concentrations of RGZ and GW9662 for in vitro treatment were determined by treating HTR8 cells for 24 h with nothing; with DMSO (volume equivalent to the maximum drug volume); or with 1, 5, 10, or 20 µm RGZ or GW9662. Changes in PPARγ activity, as determined by western blotting, were used as a basis for determining the optimal drug concentration (as detailed in Figure S2I,J, Supporting Information).

### Exosome Isolation

Human or mouse placental tissues (100 mg) were homogenized in 1 ml of lysis buffer (Thermo Pierce Biotechnology, USA) and immediately diluted tenfold with phosphate‐buffered saline (PBS) after homogenization (Figure S4G, Supporting Information). The diluted lysate was centrifuged at 300 × g for 10 min at 4 °C to remove unhomogenized tissue and cells. The mixture was subsequently centrifuged at 2000 × g for 10 min, 5000 × g for 30 min, and 10 000 × g for 30 min at 4 °C to remove dead cells and cell debris. The supernatant was subsequently centrifuged at 100 000 × g with a SW32Ti rotor (Beckman Coulter) for 1 h at 4 °C to pellet the exosomes, which were subsequently washed with PBS and filtered through a 0.22‐µm filter before centrifugation at 100 000 × g for 2 h at 4 °C to pellet them again.^[^ ^25^ ^]^ For isolation of the exosomes from the cell supernatant, the cells were seeded in 100‐mm tissue culture dishes (Biofil) in the corresponding complete culture medium. After the cells reached 80% confluence, the medium was removed and replaced with 10 ml of the same medium prepared with exosome‐depleted FBS obtained by ultracentrifugation for 16 h at 160 000 × g and 4 °C prior to filtration through a 0.22‐µm filter. The supernatant was collected 24 h later, and the exosomes were isolated with Total Exosome Isolation Reagent (Invitrogen, 4478359, USA) according to the manufacturer's instructions.^[^ ^61^ ^]^

### Exosomal Cargo Separation

The RNA cargo of HTR8‐Exos isolated from cultured cells was separated with an exoRNeasy Midi Kit (QIAGEN, 77144, The Netherlands) according to the manufacturer's instructions. For removal of exosomal RNA, intact exosomes were subjected to ultrasonic homogenization (SCIENTZ‐IID, China) on ice (45 W for 10 cycles; on for 2 s and off for 1 s). Then, RNase (Invitrogen, USA) was added, and the mixture was incubated at 37 °C for 30 min.

### Morphological Analyses—Morphological Analysis of Exosomes via Transmission Electron Microscopy (TEM)

Exosomes were placed on Formvar‐carbon‐coated EM grids after being suspended in 2% PFA. Then, 1% glutaraldehyde was used to fix the exosomes on the grids again. Next, the grids were transferred to uranyl oxalate solution for 5 min and to methyl cellulose‐UA for 10 min.^[^ ^62^ ^]^ Finally, the size and morphology of the exosomes were evaluated via TEM (JEOL JEM‐1400PLUS, Japan).

### Nanoparticle Tracking Analysis (NTA) of Exosomes

Exosomes were resuspended in PBS for subsequent particle size and quantity measurements via electrophoresis and Brownian motion video analysis via laser scattering microscopy with a ZetaVIEW Particle Metrix analyzer (ZetaVIEW S/N 17–315, Germany). At least 1000 particles in each sample were counted and analyzed (Camera 0.743 µm px^−1^, software ZetaView 8.04.02 SP2, Germany).

### Coculture Experiments

*A*s shown in Figure S2A (Supporting Information), 1–2 × 10^5^ 3T3‐L1 preadipocytes were seeded in one well of a 6‐well tissue culture plate. Approximately 2 × 10^5^ HTR8s, HUVECs, HUM cells, or WISH cells were seeded in the upper chamber (Costar, USA), which contained a 0.4 µm pore size, at the beginning of the differentiation process. The medium was changed every other day, and the cells were passaged every four days during the differentiation process. In the coculture system, which involved lentivirally transduced Tet‐shPPARγ, Tet‐NC 3T3‐L1, or HTR8 cells, the cells were studied after treatment with 1.5 µg ml^−1^ DOX for 48 h and in the presence of 1.5 µg ml^−1^ DOX throughout the differentiation process. HTR8 cells were treated with 10 µm RGZ, 10 µm GW9662, or both (10 µm GW9662 was added 2 h before the addition of 10 µm RGZ) in the insert during the differentiation process.

For the establishment of the cell‐exo coculture system, 1—2 × 10^5^ 3T3‐L1 preadipocytes were seeded in a cell culture plate. Exos containing 50 µg of total protein^[^ ^25^ ^]^ were isolated from the HTR8 culture supernatant (Exo‐depleted FBS) and added to 3T3‐L1 cells every other day, and the medium was changed for 3T3‐L1 cell differentiation. Tet‐shPPARγ or Tet‐NC HTR8 cells were treated with 1.5 µg ml^−1^ DOX for 48 h before exosome preparation. Tet‐shPPARγ or Tet‐NC 3T3‐L1 cells were treated with 1.5 µg ml^−1^ DOX for 48 h before being cocultured with Exos in the presence of 1.5 µg ml^−1^ DOX throughout the differentiation process. HTR8 cells were treated with 10 µm RGZ, 10 µm GW9662, or both (10 µm GW9662 was added 2 h before the addition of 10 µm RGZ) for 48 h before exosome preparation.

### siRNA Transfection

The siRNAs used in this study were designed and synthesized by GenePharma (Shanghai, China). The cells were seeded in 6‐well plates at a density of 1 × 10^5^ cells well^−1^ and transfected with 10 nm si‐scramble, si‐*Arid5b*, si‐*Htr2c*, si‐*Pank2*, or si‐*Rorb* with Lipofectamine 2000 (Invitrogen, USA) according to the manufacturer's recommendations. At 24 h post‐transfection, the cells were harvested and processed for qRT–PCR to verify the knockdown efficacy. The siRNA sequences used were as follows: si‐*Arid5b*, 5'‐GCCGAAGAAUAACCACAAUTT‐3'; si‐*Rorb*, 5'‐GGCAGAGAAACUGUUUAAUTT‐3'; si‐Pank2, 5'‐GGAGGUGGAUCAUACAAAUTT‐3'; si‐Htr2c, 5'‐GCCAUCGUUUGGGCAAUAUTT‐3'; and si‐scramble, 5'‐UUCUCCGAACGUGUCACGUTT‐3'.

### Induction of Differentiation in 3T3‐L1 Cells with Arid5b, Htr2c, Pank2 or Rorb Knockdown

In the 3T3‐L1 cell and HTR8‐Exo coculture system, 1–2 × 10^5^ 3T3‐L1 preadipocytes were seeded in a cell culture plate. Exos (50 µg of protein) isolated from HTR8 culture supernatant (Exo‐depleted FBS) were added to 3T3‐L1 cells every other day, and the medium was changed for 3T3‐L1 cell differentiation. During the differentiation process, 3T3‐L1 cells were treated with 10 nm si‐scramble, si‐*Arid5b*, si‐*Htr2c*, si‐*Pank2*, si‐*Rorb*, or a mixture of si‐*Arid5b*, si‐*Htr2c*, si‐*Pank2*, and si‐*Rorb* (10 nm) 2 h before the addition of HTR8‐Exos on differentiation days 0, 2, 4, and 6.

### Exosome Internalization Assay

Approximately 5 × 10^4^ 3T3‐L1 cells were seeded and allowed to adhere to a confocal dish. HTR8‐Exos containing 50 µg of protein were stained with 4 µl of PKH67 (Sigma–Aldrich, Germany), which was dissolved in 1 ml of diluent C (Sigma–Aldrich, Germany), and then centrifuged at 100 000 × g for 1 h at 4 °C to obtain PKH67‐labeled HTR8‐Exos. 3T3‐L1 cells were subsequently treated with PKH67‐labeled HTR8‐Exos or an equal volume of PKH67‐labeled exosome‐depleted supernatant for 2 h at 4 or 37 °C, followed by three washes with PBS (Figure S3G, Supporting Information). Images were then acquired with a confocal microscope (Leica, Germany). For analysis of the endocytosis of HTR8‐Exos by 3T3‐L1 cells, 3T3‐L1 cells were treated with chlorpromazine (20 µm, Selleck, USA), nystatin (54 µm, Selleck, USA), or nocodazole (1 µm, Sigma–Aldrich, Germany) for 2 h prior to another 2 h of coincubation with PKH67‐labeled HTR8‐Exos.^[^ ^25^ ^]^

### ChIP‐Seq Analysis

ChIP was performed with a commercially available kit (#9003, Cell Signaling Technology, USA) according to the manufacturer's instructions and a previous study.^[^ ^63^ ^]^ In brief, 3T3‐L1 preadipocytes and PPARγ‐OE HTR8‐Exo‐treated 3T3‐L1 preadipocytes (day 4 of differentiation) were fixed with 37% formaldehyde, pelleted, and resuspended in lysis buffer. The nuclei were then pelleted, subjected to enzymolysis, sonicated, and centrifuged to remove insoluble material. The supernatants were collected, and protein G magnetic beads (Bio‐Rad, USA) conjugated to an anti‐PPARγ antibody (Cell Signaling Technology, CST, USA) or Profinity IMAC resin (Bio‐Rad, USA) were added prior to incubation at 4 °C overnight. The bound chromatin was collected, eluted, and decrosslinked at 65 °C overnight. The DNA was then purified with a spin column, quantified with a Qubit 3.0 fluorometer (Life Technologies, USA), and assessed for integrity via agarose gel electrophoresis. Clustering of the index‐coded samples was performed on a cBot Cluster Generation System with a TruSeq PE Cluster Kit v3‐cBot‐HS (PE‐401–3001, Illumina, USA) according to the manufacturer's instructions. After cluster generation, ChIP‐seq libraries were prepared and sequenced on an Illumina HiSeq platform (NovaSeq 6000, Illumina, USA), and 50‐bp single‐end reads or 150‐bp paired‐end reads were generated. BigWig files were generated from the alignment results for visualization purposes. Adapter sequences, poly‐N sequences, and low‐quality reads were removed from the raw sequencing data. The obtained clean reads were aligned to the reference genome with BWA mem v 0.7.12 software. Model‐based analysis of ChIP‐seq (MACS2) software was used to identify peaks at the whole‐genome scale, with *p* ≤ 0.05 as the threshold criterion. For the identification of possible binding motifs of the PPARγ DNA‐binding response regulator, the sequence motifs corresponding to the ChIP peaks were analyzed with HOMER to detect long and short consensus sequences. After motif detection, HOMER was used to annotate the motifs on the basis of sequence similarity.^[^ ^64^ ^]^ Peak‐related genes were confirmed with Peak Annotator, and GO enrichment analysis was subsequently performed to examine functional enrichment. GO terms with corrected p values of less than 0.05 were considered significantly enriched in peak‐related genes. KOBAS software was used to test the statistical enrichment of peak‐related genes in KEGG pathways. A peak was considered a differential peak when the fold enrichment of the peak between the two groups was greater than 2. The ChIP‐seq data are available at https://www.ncbi.nlm.nih.gov/geo/query/acc.cgi?acc=GSE185779.

### Luciferase Reporter Assay

The binding of PPARγ to the promoters of *Arid5b, Rorb, Pank2*, and *Htr2c* was evaluated with a commercial luciferase reporter assay kit (Promega, Madison, WI, USA). In brief, a fragment of the coding sequence (CDS) of PPARγ was cloned and inserted into the pcDNA3.1(+) vector, and fragments containing the promoters of *Arid5b, Rorb, Pank2*, and *Htr2c* were cloned and inserted into the PGL3‐basic vector (Invitrogen, USA). Then, the HEK293 cells were plated in a 24‐well plate at a density of 10 000 cells well^−1^. The cells were cotransfected with the PPARγ fragment and each separate promoter fragment with Lipofectamine 3000 (Invitrogen, USA) according to the manufacturer's instructions. After 48 h, the cells were lysed and collected. Finally, the fluorescence intensity was measured with a Spark Multimode Microplate Reader (Tecan, Switzerland). The experiment was performed in triplicate.

### Proteomic Analysis of Human Placenta‐Derived Exosomes

For proteomic analysis, human placenta‐derived exosomes were first isolated via ultracentrifugation as previously described. Then, exosomal proteins were extracted with lysis buffer (Thermo Pierce Biotechnology, USA), digested with trypsin (Promega, USA), and analyzed by LC–MS/MS (nanoLC‐QE). The raw data were searched against the human UniProt database with Mascot 2.2.

### Animals

Eight‐week‐old C57BL/6 mice were purchased from the Laboratory Animal Center of Chongqing Medical University. GD 0.5 was defined as the day of conception when a vaginal plug was detected. All the mice were housed in a temperature‐controlled room (23 °C) with a 12‐h:12‐h light: dark cycle and ad libitum access to water and food. All animal experiments referred to in this study were carried out in accordance with the National Institutes of Health Guide for the Care and Use of Laboratory Animals and were approved by the Institutional Animal Care and Use Committee of Chongqing Medical University. On GD 18.5, pregnant mice were sacrificed in a CO_2_ chamber unless specifically noted otherwise. The morphology and number of offspring were recorded, and related samples were collected for subsequent use. In the plCSA‐RNP/plCSA‐GNP cotreatment group, plCSA‐GNPs were administered 2 h before plCSA‐RNPs.

### Establishment of the RUPP‐Induced Mouse Model of FGR

Pregnant C57BL/6 mice (GD 13.5) were randomly assigned to the RUPP surgery, sham surgery, or control group. RUPP surgery was performed as described previously.^[^ ^65^ ^]^ Specifically, pregnant mice were anesthetized on a 37 °C heating pad using isoflurane (Friends Honesty Life Sciences Company Limited, China) with an animal anesthesia apparatus (#Table Top 723012, Surjivet, USA) and a coupled rodent ventilator (INSPIRA ASV, Harvard Apparatus, USA). A 1‐cm abdominal incision was made along the linea alba extending from the skin to the peritoneum under aseptic conditions. A silver clip (200 µm) was then placed around the abdominal aorta below the renal arteries. Silver clips (80 µm) were also placed on the right and left uterine arcades at the ovarian end before the first segmental artery (Figure S7A, Supporting Information). Finally, the incisions in the peritoneum and skin were sutured sequentially with 7–0 nylon sutures (Lingqiao, Ningbo, China). No clips were used in the sham surgery.

### Establishment of the mouse model of LPD‐induced FGR

Eight‐week‐old pregnant C57BL/6 mice weighing 18–22 g were fed either an LPD (Research Diets, D02041002) (containing 9% protein by weight) or an isocaloric control diet (Moldiets, M19042601) (containing 19% protein by weight) from GD 0.5 to GD 18.5.^[^ ^66^ ^]^

### Generation of Mice with Placenta‐Specific PPARγ Deletion

To generate mice with placenta‐specific PPARγ deletion, DOX‐inducible *ADACre^+/+^* C57BL/6 mice were first generated (Figure 2A). Specifically, the sequence encoding Cre recombinase controlled by a Tet‐on system driven by an ADA basal promoter and ADA/4311 placenta‐specific enhancers^[^ ^19^ ^]^ was inserted into the murine EGE‐LS‐097(H11) locus via a CRISPR–Cas9/sgRNA plasmid through zygote microinjection. The target mice were obtained after the founder mice were bred and genotyped. *PPARγ^flox/flox^* C57BL/6 mice obtained from The Jackson Laboratory were crossed with *ADACre^+/+^* mice to generate *ADACre^+/−^*; *PPARγ^flox/−^* males. Next, the *ADACre^+/−^*; *PPARγ^flox/−^* males were crossed with *PPARγ^flox/flox^* females, and the pregnant mice were subsequently treated with 0.5 mg ml^−1^ DOX in the drinking water from GD13.5 to GD18.5 to obtain pregnant mice with placenta‐specific PPARγ deletion^[^ ^67^ ^]^ (Figure 2B).

### In Vivo Pharmacokinetic Analysis of plCSA‐RNPs

For analysis of the metabolism of RGZ in pregnant mice, pregnant mice (GD 13.5) were treated with RGZ dissolved in DMSO (RGZ‐DMSO) via tail vein injection. Fundus venous plexus blood was collected at 5, 15, 30 min, 1, 2, 4, 6, 8, 12, and 24 h after injection. Similarly, to determine the pharmacokinetics of plCSA‐RNPs, pregnant mice (GD 13.5) were treated with 1 or 0.1 mg kg^−1^ RGZ‐DMSO, plCSA‐RNPs, or RNPs via tail vein injection, and heart blood was collected immediately after anesthetization with isoflurane at 4, 12, 24, 36, and 48 h after injection. The RGZ concentrations in mouse fundus venous plexus blood and heart blood were then measured via liquid chromatography–mass spectrometry (LC–MS; Agilent, Canada). Specifically, after incubation for 1 h at 4 °C, the blood was centrifuged at 3000 rpm for 15 min to obtain the serum. Next, 5 µl of serum was added to 60 µl of acetonitrile (Merck, Germany) containing 10 µg ml^−1^ nonadecanoic acid (Sigma–Aldrich, Germany), and the sample was mixed by vortexing and then centrifuged at 10 000 rpm for 5 min at 4 °C. Finally, the supernatant was collected with a glass insert in a sample vial for LC–MS analysis. Data analysis was performed with MassLynx 4.0 software (Waters Corporation, USA).

### Mouse Body Composition Measurement

For DEXA scans, GD 18.5 mouse fetuses were positioned centrally at the bottom of a square and then scanned with a fan beam QDR 4500 A densitometer (Hologic, Inc., Bedford, MA, USA), which was calibrated daily in accordance with the manufacturer's recommendations. All DEXA measurements and analyses were performed by the same investigator, and the data were analyzed with the small animal mode of Hologic Discovery Software (Hologic, Inc., Bedford, USA).

### Alizarin Red/Alcian Blue Staining of Embryonic Skeletons

Alizarin red/Alcian blue double‐staining was conducted as previously described, with modifications.^[^ ^68^ ^]^ In brief, GD18.5 fetuses were fixed with 10% formalin buffer overnight, and the skin and viscera were then carefully removed. Next, fetal lipids were removed in acetone overnight and stained in staining solution (0.03% Alcian blue 8GX (Sigma–Aldrich, Germany), 0.006% Alizarin red (Sigma–Aldrich, Germany), and 10% acetic acid (Sigma–Aldrich, Germany) dissolved in 95% ethanol) for 72 h after fixation with 95% ethanol overnight. Soft fetal tissues were subsequently cleared in 1% KOH until bone (red) and cartilage (blue) could be distinctly observed. All the above procedures were conducted on a slow rocker (45–55 rpm) at room temperature. Finally, well‐stained pups were stored in glycerin after daily treatment with increasing ratios of glycerin/potassium hydroxide and were imaged via stereoscopic microscopy (Leica, Germany).

### Measurement of BP

As described previously,^[^ ^69^ ^]^ the arterial BP of conscious pregnant mice was noninvasively measured every other day from GD1.5 to GD13.5 and every day from GD14.5 to GD 18.5 (between 10:00 A.M. and noon) by determining the tail blood volume via occlusion tail‐cuff plethysmography with a volume pressure recording sensor (BP‐2000 Blood Pressure Analysis System, Visitech Systems, USA). The mice were trained to adapt to the apparatus for 7 consecutive days. Actual BP measurements were initiated after the mouse was maintained in the restrainers for 5 to 10 preliminary measurements, and the systolic and diastolic BP values were calculated by averaging 10 to 20 actual BP measurements obtained in each session.

### Enzyme‐Linked Immunosorbent Assay (ELISA)

The concentration of sFlt‐1 in the peripheral blood of GD 18.5 pregnant mice was measured with commercial ELISA kits (Elabscience, Wuhan, China) in accordance with the manufacturer's instructions. In brief, the microplates were coated with anti‐sFlt‐1 antibodies at 4 °C overnight. The standard and samples were subsequently incubated with HRP‐conjugated anti‐sFlt‐1 antibodies at 37 °C for 90 min. After thorough washing, tetramethylbenzidine (TMB) substrate was added for colorimetric measurement of the concentrations in the samples. The optical density (OD) at 450 nm was measured with a microplate reader (Thermo Fisher, USA), and the concentration of sFlt‐1 in each sample was calculated on the basis of the standard curve.

### Lipid Staining

Lipid accumulation in frozen tissue sections and differentiated 3T3‐L1 cells was detected via oil red O staining (Sigma–Aldrich, Germany). The samples were washed three times with PBS, fixed with 4% PFA for 1 h at room temperature, rinsed with PBS twice, and stained with filtered oil red O solution (0.5 g in 100 ml of isopropyl alcohol) for 30 min. The samples were then washed twice with distilled water and viewed and photographed with a microscope (EVOS, Life Science, USA).^[^ ^70^ ^]^ The oil‐red O‐stained areas were analyzed by ImageJ.

### Periodic Acid–Schiff (PAS) Staining

Kidney tissue was fixed with 10% neutral formalin, embedded in paraffin, and sliced into 5 µm thick sections. After deparaffinization, the sections were subjected to PAS staining to detect glycogen accumulation in the glomerulus.^[^ ^65^ ^]^

### Hematoxylin‒Eosin (H&E) Staining

In brief, tissue samples were sliced into 5‐µm sections after being fixed with 10% neutral formalin and embedded in paraffin. After deparaffinization and rehydration, the sections were stained with hematoxylin solution for 5 min, dipped 5 times into 1% acid ethanol (1% HCl in 70% ethanol), and then rinsed in distilled water. The sections were subsequently stained with eosin solution for 3 min, dehydrated through a graded alcohol series, and cleared in xylene. The mounted slides were then photographed with a fluorescence microscope (EVOS, Life Science, USA).

### Analysis of Metabolic Parameters

After homogenization, 50 mg of GD18.5 fetal liver tissue from each group was used to measure the total cholesterol and TG contents with commercially available assay kits (Tissue Total Cholesterol Assay Kit E1015 and Tissue Triglyceride Assay Kit E1013, Applygen Technologies, Beijing, China) according to the manufacturer's instructions. The GD 18 dams were fasted overnight with free access to tap water, tail vein blood was collected the next day at 8:00 A.M., and the serum concentration of glucose was measured with a commercially available assay kit (Glucose Oxidase Method E1010, Applygen Technologies, Beijing, China).

### Immunoblot Analysis

Total protein was extracted with lysis buffer and quantified with a BCA protein assay kit (Thermo Pierce Biotechnology, USA) according to the manufacturer's instructions. Proteins were then separated by electrophoresis at 120 V for 120 min and transferred onto PVDF membranes (pore size: 0.45 µm, Millipore, Germany) at 100 V for 120 min. Subsequently, the membranes were blocked with 5% nonfat dried milk powder (Bio‐Rad, USA) and then incubated overnight at 4 °C with the following primary antibodies: anti‐PPARγ (Santa Cruz Biotechnology, sc‐7196, USA; diluted 1:1000); anti‐p‐PPARγ^S112^ (Invitrogen, PA5‐36763, USA; diluted 1:500); anti‐p‐PPARγ^S273^ (Bioss, bs‐4888R, China; diluted 1:1000); anti‐PLAP (Proteintech, 18507‐1‐AP, China; diluted 1:1000); anti‐ACC (Cell Signaling Technology, 3662, USA; diluted 1:1000); anti‐p‐ACC^S79^ (Cell Signaling Technology, 3661, USA; diluted 1:1000); anti‐SIRT1 (Cell Signaling Technology, 8469, USA; diluted 1:1000); anti‐PGC‐1α (Cell Signaling Technology, 2178, USA; diluted 1:1000); and anti‐tubulin (Cell Signaling Technology, 2148, USA; diluted 1:1000) antibodies; or anti‐SREBP1 (Abcam, ab28481, UK; diluted 1:1000), anti‐C/EBPα (Abcam, ab140479, UK; diluted 1:1000), anti‐CD9 (Abcam, ab223052, UK; diluted 1:1000), anti‐CD63 (Abcam, ab213090, UK; diluted 1:1000), anti‐CD81 (Abcam, ab109201, UK; diluted 1:1000), anti‐Alix (Abcam, ab275377, UK; diluted 1:1000), anti‐HSP90 β (Abcam, ab203085, UK; diluted 1:1000), anti‐Integrin α5 (Abcam, ab150361, UK; diluted 1:1000), and anti‐TSG101 (Abcam, ab125011, UK; diluted 1:1000) antibodies. The membranes were then washed and incubated with horseradish peroxidase‐conjugated anti‐rabbit or anti‐mouse secondary antibodies (Santa Cruz Biotechnology, USA; diluted 1:5000) for 1 h at room temperature. Finally, the membranes were scanned with a Bio‐Rad XRS+ imaging system using enhanced chemiluminescence reagents (Advansta, USA), and expression was analyzed with Bio‐Rad Image Lab 5.2 software. The primary antibodies were diluted in 5% bovine serum albumin (BSA) in 1× Tris‐buffered saline (TBS) containing 0.1% Tween 20.

### qRT–PCR

Total RNA was extracted with TRIzol reagent (Invitrogen, USA), and a Transcriptor First Strand cDNA Synthesis Kit (Roche, Germany) was subsequently used to synthesize cDNA according to the manufacturer's instructions. qRT–PCR was performed with FastStart Essential DNA Green Master Mix (Roche, Germany) in a real‐time system (Bio‐Rad CFX96). The thermal cycling conditions used for qRT‒PCR were as follows: 10 min at 95 °C for polymerase activation and cDNA denaturation; 40 cycles of 10 s at 95 °C, 30 s at the optimum temperature for the primers, and 10 s at 72 °C for amplification; and 0.5 °C increments from 65 to 95 °C every 5 s for melt curve analysis. The results were analyzed with Bio‐Rad CFX Maestro 1.1 software. In brief, the abundance of each target mRNA was determined on the basis of the cycle threshold value. The relative changes in gene expression were determined via normalization to the expression of the housekeeping gene β‐actin or ribosomal phosphoprotein 0 (RPLP0). The sequences of primers used for amplification of the target genes are listed in Table S5 (Supporting Information).

### Immunofluorescence Analysis

Ten‐micrometer‐thick frozen sections were first heated at room temperature (RT) for 2 h, washed with RT‐TBS to remove the cryopreservation medium, permeabilized in 5% PBS containing 0.2% Triton X‐100, and blocked in 5% BSA containing 0.1% Triton X‐100 for 2 h at RT. The sections were subsequently incubated with primary antibodies specific for PPARγ (Santa Cruz Biotechnology, sc‐7196, USA; diluted 1:100) and CK7 (Abcam, ab181598, UK; diluted 1:100) overnight at 4 °C. The sections were then reblocked in 5% BSA containing 0.1% Triton X‐100 for 1 h at RT, incubated with HRP‐conjugated secondary antibodies (Santa Cruz Biotechnology, USA; diluted 1:200) for 2 h at RT, stained with DAPI, and mounted. Finally, the sections were viewed with a confocal fluorescence microscope (Leica, Germany).

### Statistical Analysis

Differences in fetal sex and neonatal intensive care unit occupancy rates between the FGR and normal groups were calculated via Pearson's *x* ^2^ test and Fisher's exact test, respectively, with SPSS 22.0. Unpaired two‐tailed Student's *t*‐test (two groups) and nonparametric one‐way ANOVA (multiple groups) were also used to identify differences with GraphPad Prism 7.0. All the results were presented as the means ± SDs. The figure legends indicated the number of independent experiments performed in each analysis. Significant differences were defined as those for which *P* < 0.05. Unless otherwise noted, each experiment was performed at least two times.

## Conflict of Interest

The authors declare no conflict of interest.

## Author contributions

X.L. and B.H. contributed equally to this work. C.T. and H.Q. conceived and designed the study. X.L., B.H., and P.X. established the animal models. B.Z. and X.F. synthesized the nanoparticles. X.L., B.H., H.W., L.L., J.L., M.H., X.L., and J.H. performed the experiments and analyzed the data. M.K., R.K., and Y.X. interpreted the results. X.L., B.H., and C.T. wrote the manuscript draft. L.X. and B.H. prepared the figures. Y.F., M.K., and P.B. edited the manuscript. C.T. and H.Q. provided funding. Y.X., P.B., H.Q., and C.T. cosupervised the work.

## Supporting information



Supporting Information

Supplemental Video S1

## Data Availability

The data that support the findings of this study are available from the corresponding author upon reasonable request.
