# Supplementary material for: The Placenta Regulates Intrauterine Fetal Growth via Exosomal PPARγ
Source: Adv Sci (Weinh). 2025 Feb 14;12(15):2404983. doi: 10.1002/advs.202404983 (PMC12005745; doi:10.1002/advs.202404983)
Supplement: Supplementary file 1 — Supporting Information [file ADVS-12-2404983-s002.docx]

**Supplementary Data**

**
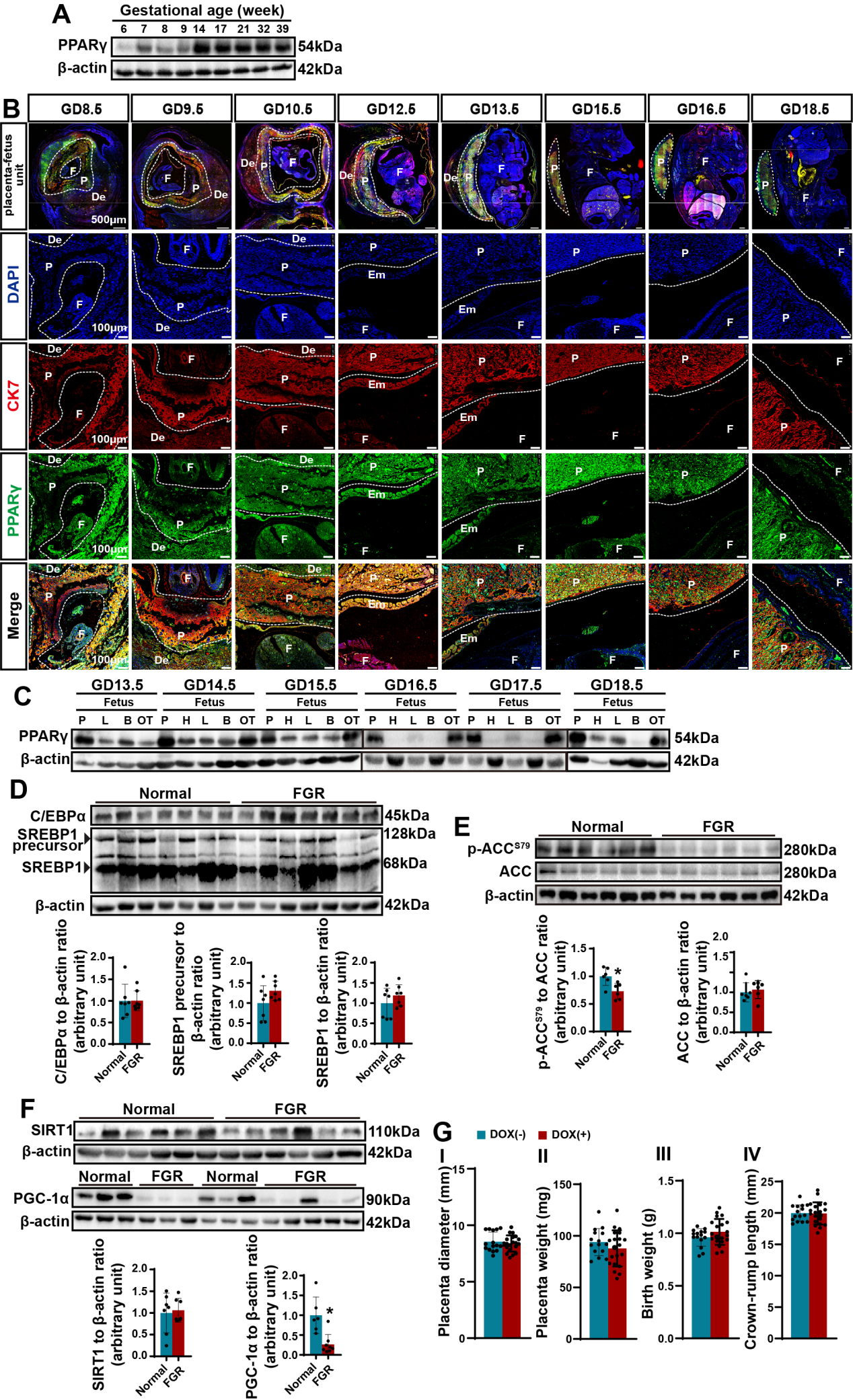
**

**Fig. S1: PPARγ expression is high in normal placentas but compromised in FGR-affected placentas.**

**A.** Immunoblot analysis of PPARγ in human placentas at different gestational ages; β-actin was used as the loading control. n=3 biological samples per group; data for one representative sample from each group are shown. **B.** Confocal micrographs of PPARγ (green) and CK7 (red) staining in frozen sections of mouse placental–fetal units collected on the indicated gestational day. Nuclei were counterstained with DAPI (blue). n=3 biological samples per group; one representative image from each group is shown. P: placenta, F: fetus, De: decidua, Em: embryo lemma. Scale bar: 500 µm (in the placental–fetal unit), Scale bar: 100 µm (in the zoomed-in fields of view). **C.** Immunoblot analysis of PPARγ expression in the placenta (P), fetal heart (H), fetal liver (L), fetal brain (B) and organ-depleted tissues (OT, fetal mouse tissue after removal of the cranial, chest and enterocele contents) of mice collected on the indicated gestational days. n=3 biological samples per group; data for one representative sample from each group are shown. **D.** Immunoblot analysis of C/EBPα, SREBP1 precursor, and SREBP1 in human normal and FGR placentas. n=7 biological samples per group. Student’s t test. **E.** Immunoblot analysis of p-ACC^S79^ and ACC in human normal and FGR placentas. n=7 biological samples per group. Student’s t test. *P<0.05. **F.** Immunoblot analysis of SIRT1 and PGC-1α expression in human normal and FGR placentas. n=6 biological samples per group for the analysis of SIRT1; n=6 biological samples in the normal group and n=8 biological samples in the FGR group for the analysis of PGC-1α. Student’s t test. *P<0.05. **G.** Analysis of placental diameter (**I**), placental weight (**II**), fetal birth weight (**III**), and crown–rump length (**IV**) in DOX-treated wild-type [DOX(+)] and control wild-type [DOX(-)] mice at GD 18.5. The DOX was used beginning at GD 13.5. Student’s t test. *P<0.05.

**
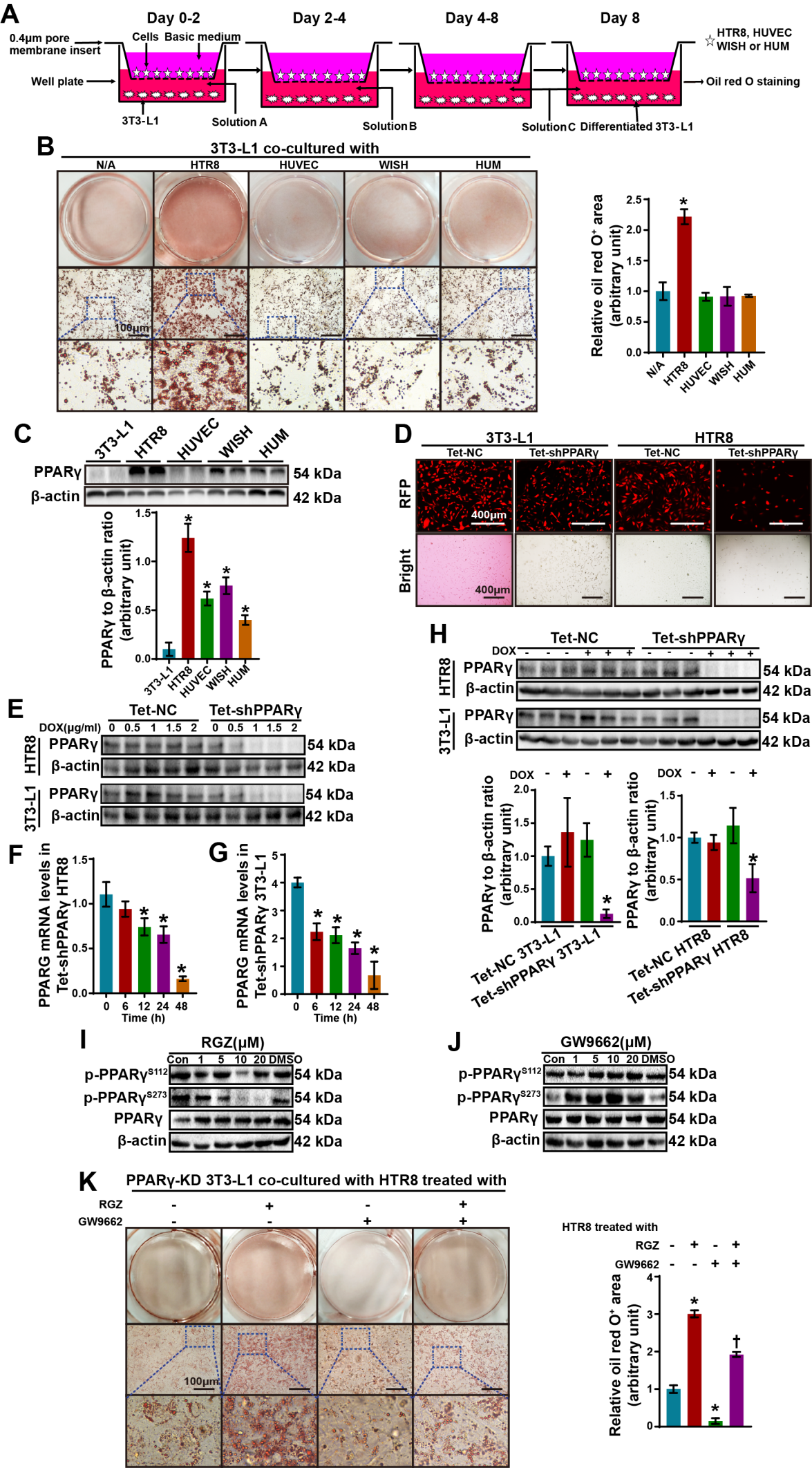
**

**Fig. S2: Establishment of conditional PPARγ-deficient HTR8 and 3T3-L1 cell lines.**

**A.** Diagram showing the procedure for coculturing 3T3-L1 and HTR8 cells in this study. **B.** Oil red O-stained 3T3-L1 cells on differentiation day 8 after culture alone or coculture with HTR8 cells, HUVECs (human umbilical vein endothelial cells), WISH (amniotic epithelial) cells, or HUM (uterine smooth muscle) cells. n=3 independent experiments; 1 representative image from each group is shown. Two-way ANOVA. *P<0.05 *vs.* N/A. Scale bar: 100 µm. **C.** Immunoblot analysis of PPARγ in 3T3-L1 cells, HTR8 cells, HUVECs, WISH cells, and HUM cells. n=4 samples per group; data for 2 representative samples per group are shown. Two-way ANOVA. *P<0.05 *vs.* 3T3-L1 cells. **D.** Fluorescence and bright field images of Tet-NC and Tet-shPPARγ lentivirus-infected 3T3-L1 and HTR8 cells treated with 1.5 µg/ml DOX for 48 h. n=3 samples per group; one representative image from each group is shown. Scale bar: 400 µm. **E.** Immunoblot analysis of PPARγ in Tet-NC and Tet-shPPARγ HTR8 and 3T3-L1 cells treated with the indicated concentrations of DOX for 48 h. This experiment was repeated three times. **F-G.** qRT–PCR analysis of *PPARG* in Tet-shPPARγ HTR8 (**F**) and Tet-shPPARγ 3T3-L1 (**G**) cells after treatment with 1.5 µg/ml DOX for the indicated durations. n=3 samples per group. One-way ANOVA. *P<0.05 *vs.* 0 h. **H.** Immunoblot analysis of PPARγ in Tet-NC, Tet-shPPARγ HTR8 and 3T3-L1 cells after treatment with 1.5 µg/ml DOX for 48 h. n=3 samples per group. One-way ANOVA. *P<0.05 *vs.* Dox-untreated (DOX-) Tet-shPPARγ HTR8 or 3T3-L1 cells. **I‒J.** Immunoblot analysis of p-PPARγ^S112^, p-PPARγ^S273^, and PPARγ in HTR8 cells after treatment for 48 h with the indicated concentrations of RGZ (I) or GW9662 (**J**). DMSO was used as the vehicle. This experiment was repeated three times*.* **K.** Oil red O-stained PPARγ-KD 3T3-L1 cells on differentiation day 8 after culture with RGZ, GW9662, or both. n=3 independent experiments; 1 representative image from each group is shown. Two-way ANOVA. *P<0.05 *vs.* untreated cells, †P<0.05 *vs.* GW9662-treated cells. Scale bar: 100 µm.

**
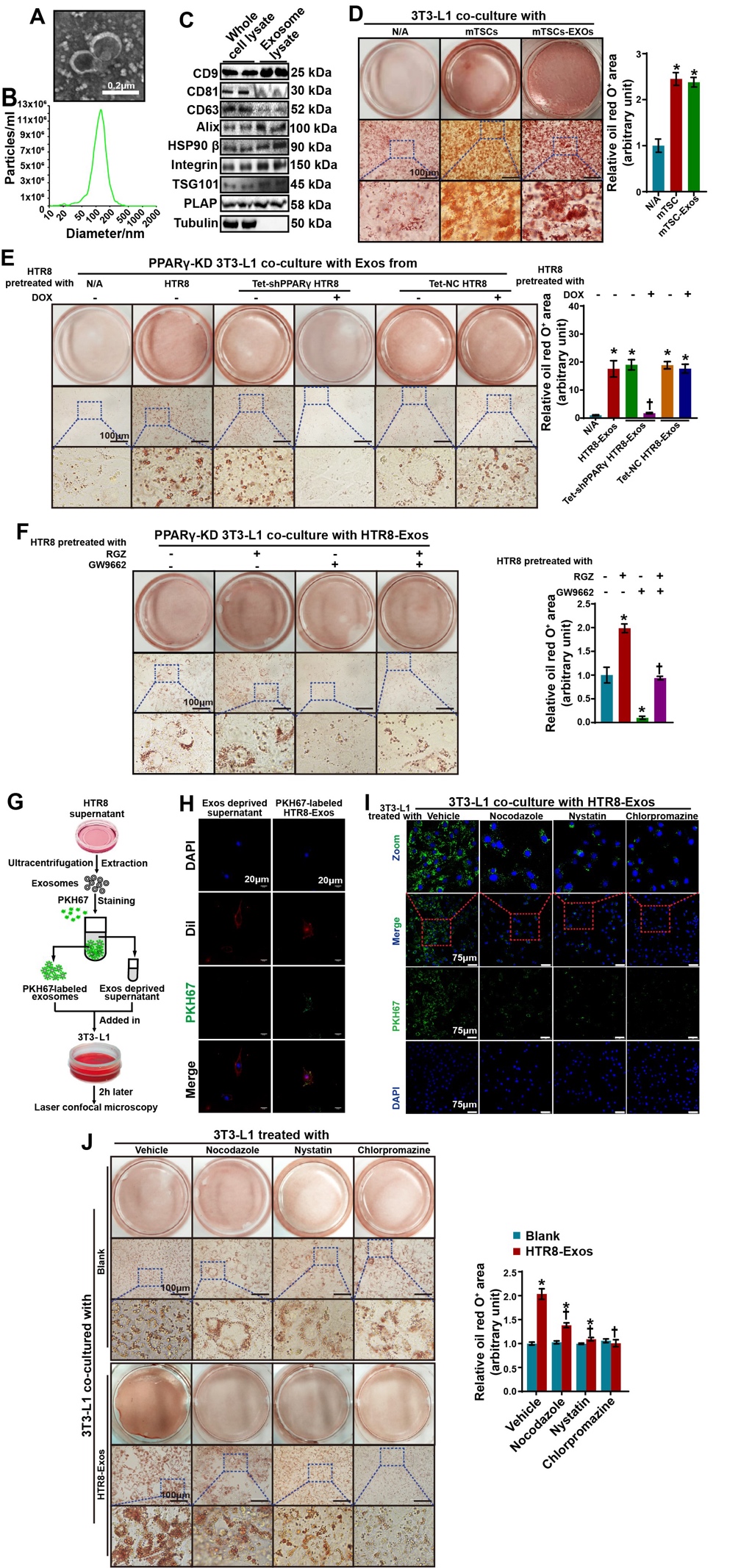
**

**Fig. S3: Transport of trophoblast-derived exosomes into preadipocytes is reduced for increased differentiation.**

**A.** Representative TEM image of HTR8-Exos. Scale bar: 0.2 µm. **B.** NTA (nanoparticle tracking analysis) of HTR8-Exos. **C**. Immunoblot analysis of CD9, CD81, CD63, Alix, HSP90β, integrin, TSG101, PLAP and tubulin in whole-cell and exosome lysates of HTR8 cells. This experiment was repeated three times. **D.** Oil red O-stained 3T3-L1 cells on differentiation day 8 after culture alone (N/A) or coculture with mouse trophoblast stem cells (mTSCs) or mTSC-derived exosomes (mTSC-EXOs). n=3 independent experiments with 3 independent preparations of exosomes. Two-way ANOVA. *P<0.05 *vs.* N/A. Scale bar: 100 µm. **E.** Oil red O-stained PPARγ-KD 3T3-L1 cells on differentiation day 8 after culture alone (N/A) or coculture with exosomes derived from the indicated HTR8 cells with/without DOX pretreatment for 48 h. n=3 independent experiments with 3 independent preparations of exosomes. Two-way ANOVA. *P<0.05 *vs.* N/A, †P<0.05 *vs.* HTR8-Exos. Scale bar: 100 µm. **F.** Oil red O-stained PPARγ-KD 3T3-L1 cells on differentiation day 8 after coculture with exosomes from HTR8 cells pretreated with RGZ, GW9662 or both. n=3 independent experiments with 3 independent preparations of exosomes. Two-way ANOVA. *P<0.05 *vs.* untreated HTR8-Exos, †P<0.05 *vs.* GW9662-treated HTR8-Exos. Scale bar: 100 µm. **G.** Experimental design for visualizing HTR8-Exo internalization in 3T3-L1 cells. **H.** Confocal micrographs of 3T3-L1 cells after treatment with PKH67-labeled HTR8-Exos or Exo-depleted supernatant for 2 h and 3 rinses with PBS. n=3 independent experiments with 3 independent preparations of exosomes. Scale bar: 20 µm. **I.** Nocodazole, nystatin or chlorpromazine was added to 3T3-L1 cells 2 h before they were cocultured with PKH67-labeled HTR8-Exos for another 2 h. n=3 independent experiments with 3 independent preparations of exosomes. Scale bar: 75 µm. **J.** Oil red O-stained 3T3-L1 cells on differentiation day 8 after coculture with or without HTR8-Exos in the presence of nocodazole, nystatin or chlorpromazine during differentiation. n=3 independent experiments with 3 independent preparations of exosomes. Two-way ANOVA. *P<0.05 *vs.* 3T3-L1 cells cultured alone with the same treatment. †P<0.05 *vs.* vehicle-treated cocultured cells. Scale bar: 100 µm.

**
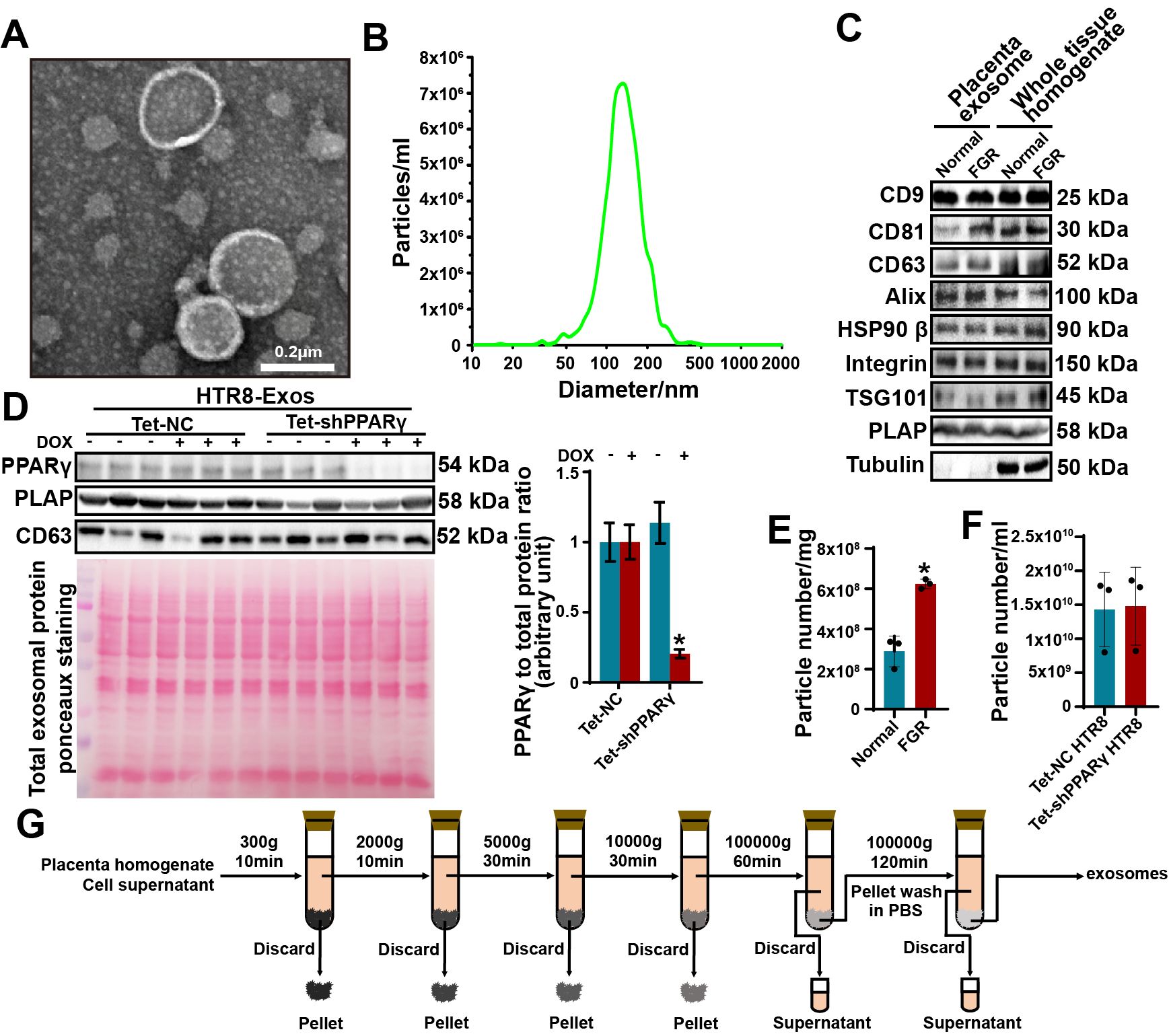
Fig. S4: Exosome isolation, verification, and cargo detection.**

**A.** Representative TEM image of exosomes isolated from human term placentas. Scale bar: 0.2 µm. **B.** NTA of exosomes isolated from human term placentas. **C.** Immunoblot analysis of CD9, CD81, CD63, Alix, HSP90β, Integrin, TSG101, PLAP and tubulin in whole homogenates and exosomes from term placentas of women with normal pregnancies and FGR-complicated pregnancies. This experiment was repeated three times. **D.** Immunoblot analysis of PPARγ, CD63 and PLAP in Tet-NC and Tet-shPPARγ HTR8-Exos. The loading amount of total protein was determined via Ponceau staining. n=3 samples per group. Two-way ANOVA. *P<0.05 compared with the DOX (-) group. **E.** Quantification of exosomes in human term placentas from women with normal and FGR-complicated pregnancies. n=3 biological samples per group. Student’s t test. *P<0.05. **F.** Quantification of exosome secretion from DOX-treated Tet-NC HTR8 cells and DOX-treated Tet-shPPARγ HTR8 cells. n=3 samples per group. Student’s t test. *P<0.05. **G.** Schematic diagram showing the process of exosome isolation by ultracentrifugation.

**
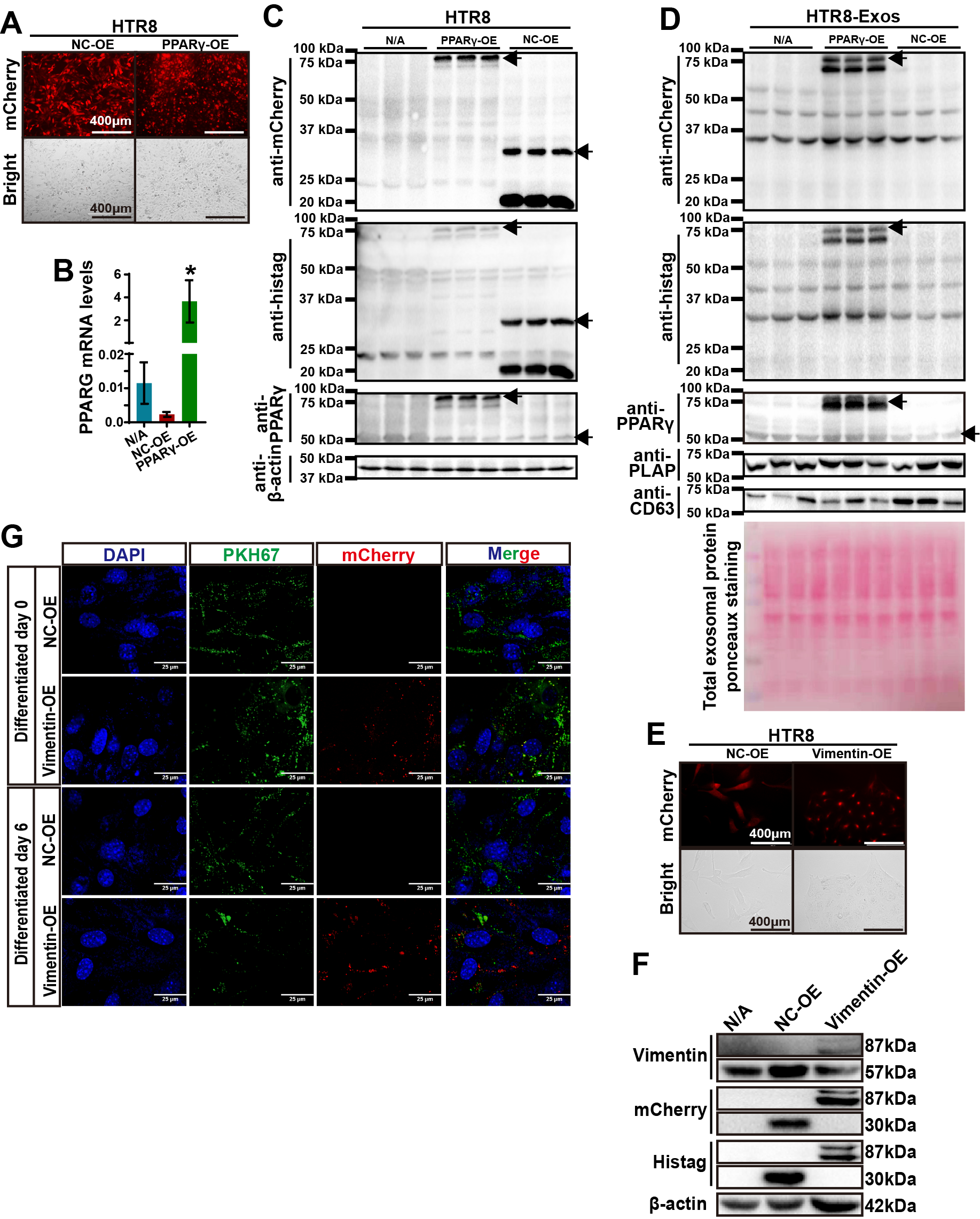
Fig. S5: Establishment of PPARγ-OE HTR8 cells.**

**A.** Fluorescence micrographs of NC-OE and PPARγ-OE lentivirus-infected HTR8 cells. RFP: red fluorescent protein. n=3 samples per group; one representative image from each group is shown. Scale bar: 400 µm. **B.** qRT‒PCR analysis of *PPARG* in nontransduced (N/A) and NC-OE or PPARγ-OE lentivirus-infected HTR8 cells. n=3 samples per group. One-way ANOVA. *P<0.05 *vs.* NC-OE. **C.** Immunoblot analysis of mCherry, His tag, and PPARγ expression in nontransduced (N/A) and PPARγ-OE or NC-OE lentivirus-infected HTR8 cells. N=3 samples per group. **D.** Immunoblot analysis of mCherry, His-tag, PPARγ, CD63 and PLAP expression in exosomes derived from nontransduced (N/A) and PPARγ-OE or NC-OE lentivirus-infected HTR8 cells. The loading amount of total protein was determined via Ponceau staining. n=3 samples per group. **E.** Fluorescence micrographs of NC-OE and Vimentin-OE lentivirus-infected HTR8 cells. n=3 samples per group; one representative image from each group is shown. Scale bar: 400 µm. **F.** Immunoblot analysis of mCherry, His tag, and Vimentin expression in nontransduced (N/A) and Vimentin-OE or NC-OE lentivirus-infected HTR8 cells. n=3 samples per group; one representative blot from each group is shown. **G.** Confocal micrographs of mCherry-fused exosomal Vimentin (red) in 3T3-L1 cells on differentiation days 0 and 6 after coculture with PKH67-labeled exosomes (green) derived from Vimentin-OE HTR8 cells. Scale bar: 25 μm. n=3 independent experiments with 3 independent preparations of exosomes.

**
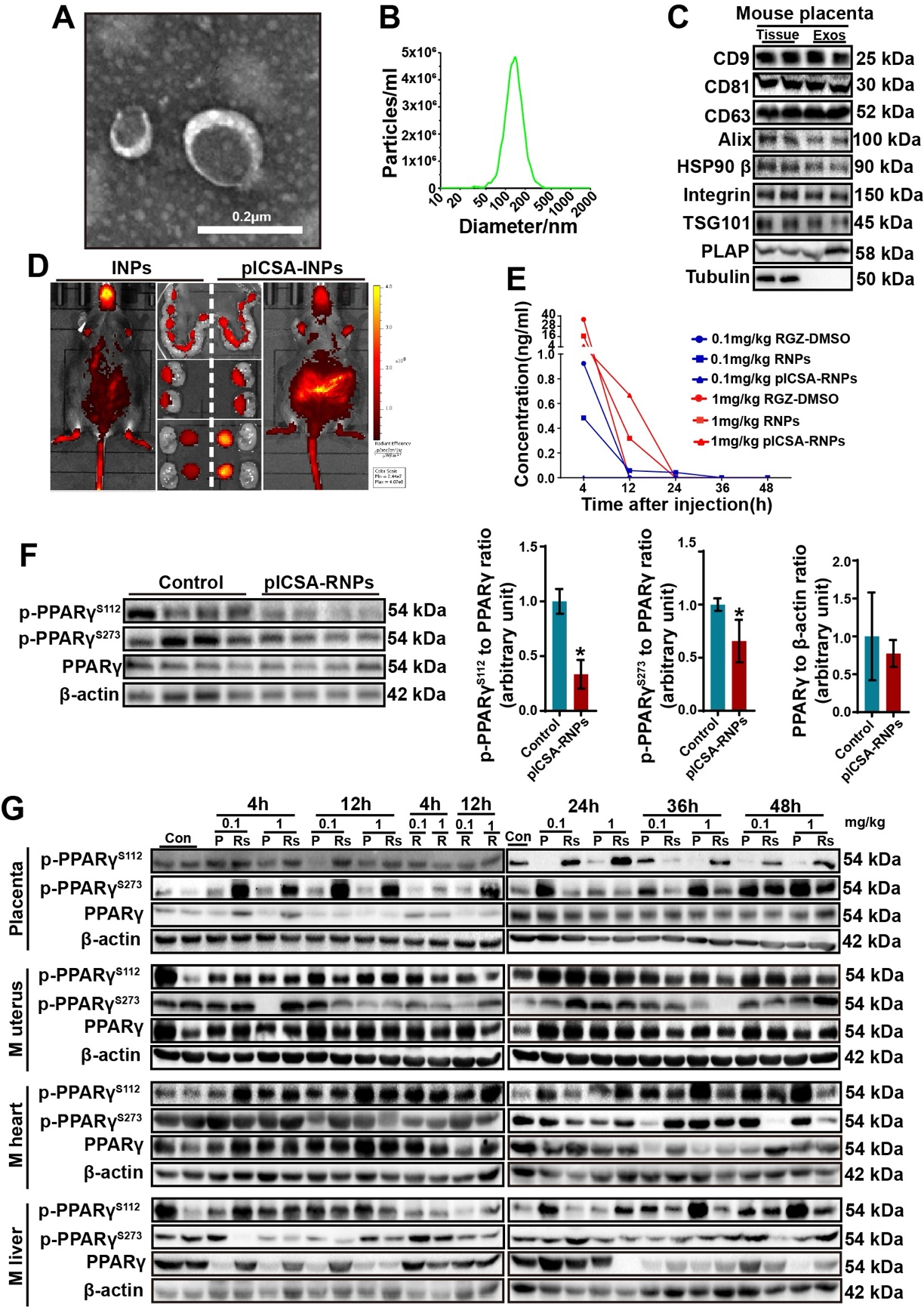
**

**Fig. S6: Specific activation of placental PPARγ in mice by plCSA-RNPs.**

**A.** Representative TEM image of exosomes isolated from GD 13.5 mouse placentas. Scale bar: 0.2 µm. **B.** NTA of exosomes isolated from GD 13.5 mouse placentas. **C.** Immunoblot analysis of CD9, CD81, CD63, Alix, HSP90β, Integrin, TSG101, PALP and tubulin in whole homogenates and exosomes isolated from GD 13.5 mouse placentas. The experiment was repeated three times. **D.** Images of pregnant mice (GD 14.5) 30 min after tail vein injection of INPs or plCSA-INPs (ICG, equivalent to 5 mg/kg), acquired with an IVIS Spectrum optical imaging system. n=3 dams per group; one representative image from each group is shown. **E.** LC–MS measurement of RGZ in the serum of GD 13.5 pregnant mice at the indicated times after injection of 0.1 mg/kg or 1 mg/kg RGZ-DMSO, RNPs, or plCSA-RNPs via the tail vein. n=4 dams per group. **F.** Immunoblot analysis of p-PPARγ^S112^, p-PPARγ^S273^, and PPARγ in GD 13.5 mouse placentas 24 h after injection with 0.1 mg/kg plCSA-RNPs via the tail vein. n=4 samples from 4 dams. Student’s t test. *P<0.05. **G.** Immunoblot analysis of p-PPARγ^S112^, p-PPARγ^S273^, and PPARγ in GD 13.5 mouse placentas and the maternal (M) uterus, heart, and liver at the indicated times after injection of 0.1 mg/kg or 1 mg/kg RGZ-DMSO (R), RNPs (Rs), or plCSA-RNPs (P) via the tail vein.

**
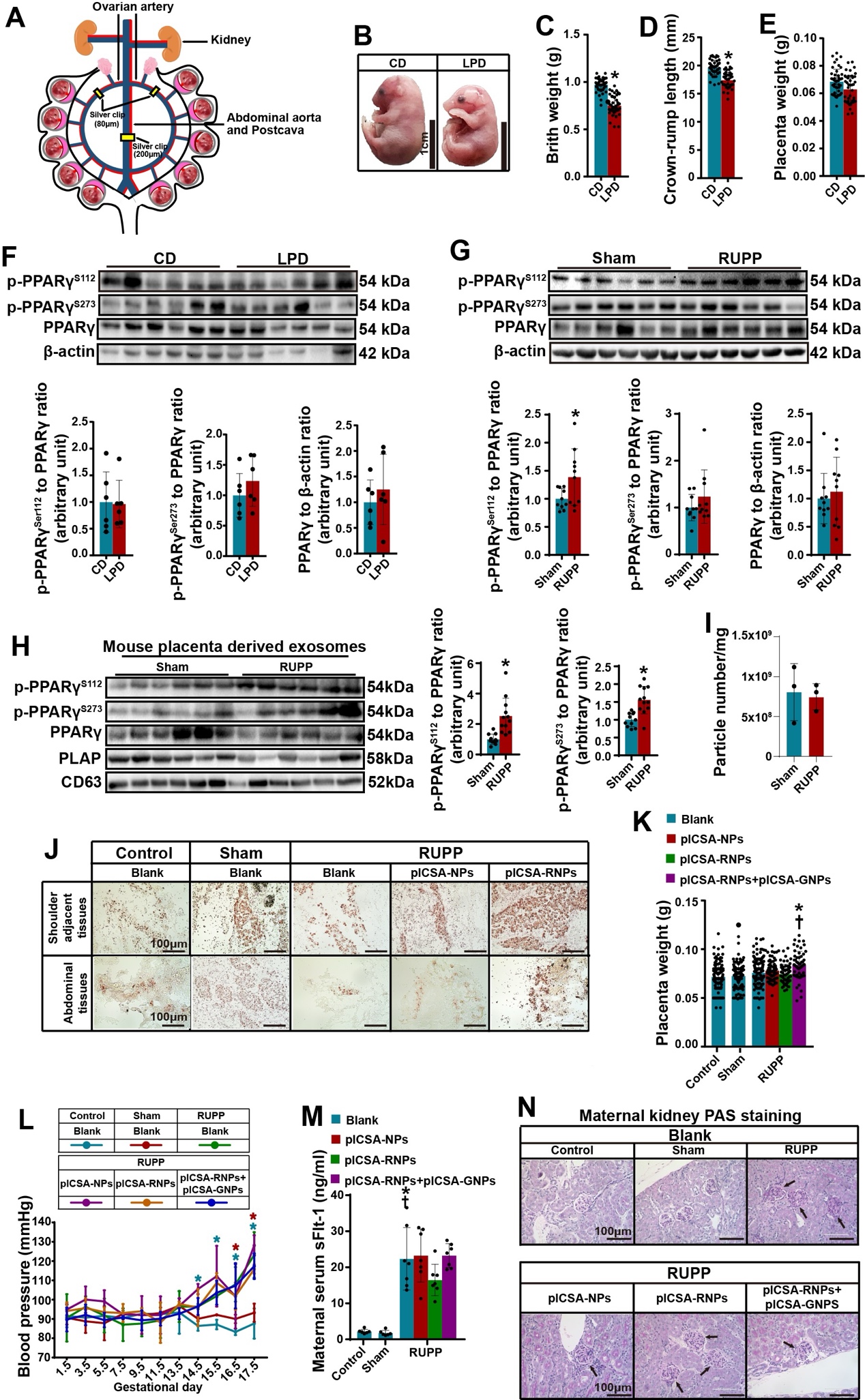
Fig. S7: Placenta-specific activation of PPARγ rescues** **RUPP-induced FGR in mice.**

**A.** Diagram of the procedure for RUPP surgery in mice. **B.** Representative images of fetuses from the CD and LPD groups. Scale bar: 1 cm. **C-E.** Fetal BW (**C**), CRL (**D**), and placental weight (**E**) in the CD and LPD groups. n=47 litters from 8 dams in the CD group and n=43 litters from 7 dams in the LPD group. Student’s t test. *P<0.05. **F.** Immunoblot analysis of p-PPARγ^S112^, p-PPARγ^S273^, and PPARγ in placentas from mice in the CD and LPD groups. n=6 samples from 6 dams per group. Student’s t test. **G.** Immunoblot analysis of p-PPARγ^S112^, p-PPARγ^S273^, and PPARγ in placentas from mice in the sham and RUPP groups. n=10 samples from 10 dams per group, and six representative samples from each group are shown. Student’s t test. *P<0.05 *vs.* sham. **H.** Immunoblot analysis of p-PPARγ^S112^, p-PPARγ^S273^, PPARγ, CD63 and PLAP in placenta-derived exosomes from mice in the sham and RUPP groups. n=11 samples from 11 dams in the sham group and 12 samples from 12 dams in the RUPP group. Six representative images from each group are shown. Student’s t test. *P<0.05 *vs.* sham. **I.** Quantification of exosomes in the placentas of the mice in the sham and RUPP groups via NTA. n=3 samples from 3 dams per group. Student’s t test. **J.** Oil red O-stained sections of abdominal tissue and shoulder-adjacent tissue from fetuses in the control, sham, and RUPP groups subjected to the indicated treatments. n=3 samples from 3 dams per group; one representative image from each group is shown. Scale bar: 100 µm. **K.** Placental weights of dams in the control, sham, and RUPP groups subjected to the indicated treatments. Control-Blank, n=150 litters from 22 dams. Sham-Blank, n=125 litters from 20 dams. RUPP-Blank, n=164 litters from 30 dams. RUPP-plCSA-NPs, n=69 litters from 12 dams. RUPP-plCSA-RNPs, n=57 litters from 12 dams. RUPP-plCSA-RNPs plus plCSA-GNPs, n=58 litters from 12 dams. Two-way ANOVA. *P<0.05 *vs.* RUPP-Blank; †P<0.05 *vs.* RUPP-plCSA-RNPs. For cotreatment with plCSA-RNPs plus plCSA-GNPs, plCSA-GNPs were given 2 h prior to plCSA-RNPs. **L.** Maternal blood pressure of the dams in the control, sham, and RUPP groups subjected to the indicated treatments. n=3 dams per group. Student’s t test. *(blue) P<0.05 *vs.* Control-Blank, *(red) P<0.05 *vs.* Sham-Blank. **M.** Serum sFlt-1 levels in dams in the control, sham, and RUPP groups subjected to the indicated treatments. Control-Blank, n=6; Sham-Blank, n=6; RUPP-Blank, n=7; RUPP-plCSA-NPs, n=7; RUPP-plCSA-RNPs, n=7; RUPP-plCSA-RNPs plus plCSA-GNPs, n=7. Two-way ANOVA. *P<0.05 *vs.* Control-Blank. †P<0.05 *vs.* Sham-Blank. **N.** PAS staining of kidneys from dams in the control, sham and RUPP groups subjected to the indicated treatments. Scale bar: 100 µm. n=6 dams per group; one representative image from each group is shown.

**
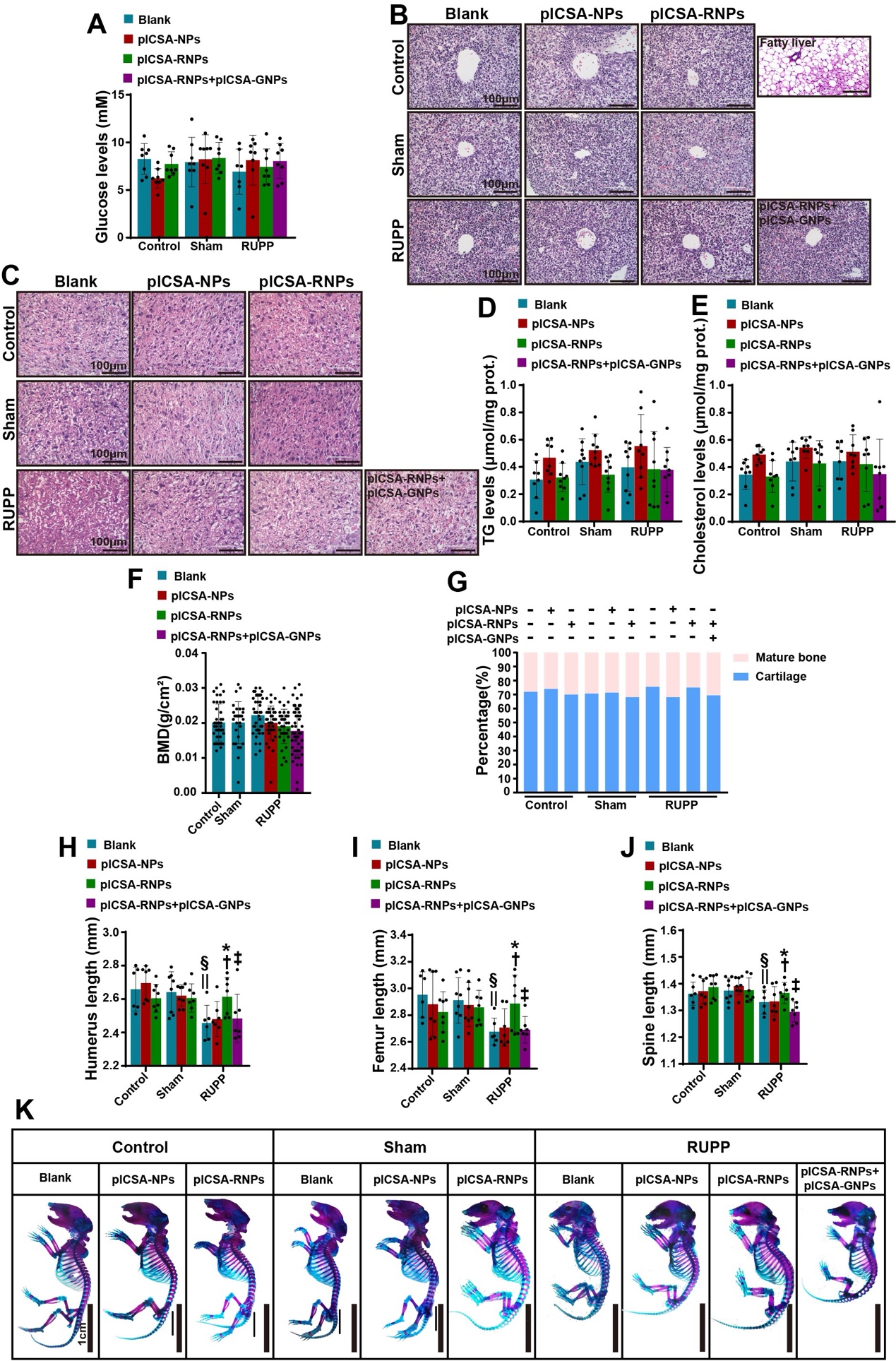
Fig. S8: Placenta-targeted delivery of RGZ does not disrupt glucose or fatty acid metabolism or bone development in mouse fetuses.**

**A.** Blood glucose levels of dams in the control, sham, and RUPP groups subjected to the indicated treatments were measured on GD 18.5; n=7 dams in the RUPP-Blank group and 8 dams in the other groups. Two-way ANOVA. **B-C.** H&E staining of the fetal liver (**B**) and placenta (**C**) of dams in the control, sham, and RUPP groups subjected to the indicated treatments. Fatty livers from mice with diet-induced obesity were used as controls. n=3 samples from 3 dams per group; one representative image from each group is shown. Scale bar: 100 µm. **D-E.** Liver triglyceride (TG) levels (**D**) and cholesterol levels (**E**) in fetuses in the control, sham, and RUPP groups subjected to the indicated treatments. Control-Blank, n=8 litters from 3 dams. Control-plCSA-NPs, n=8 litters from 3 dams. All other groups, n=9 litters from 3 dams. Two-way ANOVA. **F.** Fetal bone mineral density (BMD) analysis of dams in the control, sham, and RUPP groups subjected to the indicated treatments. Control-Blank, n=41 litters from 8 dams. Sham-Blank, n=30 litters from 7 dams. RUPP-Blank, n=39 litters from 6 dams. RUPP-plCSA-NPs, n=35 litters from 6 dams. RUPP-plCSA-RNPs, n=35 litters from 8 dams. RUPP-plCSA-RNPs plus plCSA-GNPs, n=49 litters from 7 dams. Two-way ANOVA. **G-K.** Analysis of mature bone and cartilage percentage (**G**), humerus length (**H**), femur length (**I**), and spine length (**J**) and representative images of Alizarin red/Alcian blue staining in fetuses (**K**) from dams in the control, sham, and RUPP groups subjected to the indicated treatments. Control-Blank, n=6 litters from 3 dams. Control-plCSA-NPs, n=7 litters from 3 dams. Control-plCSA-RNPs, n=8 litters from 4 dams. Sham-Blank, n=8 litters from 4 dams; Sham-plCSA-NPs, n=8 litters from 4 dams. Sham-plCSA-RNPs, n=7 litters from 3 dams. RUPP-Blank, n=6 litters from dams. RUPP-plCSA-NPs, n=7 litters from 3 dams. RUPP-plCSA-RNPs, n=7 litters from 3 dams. RUPP-plCSA-RNPs plus plCSA-GNPs, n=7 litters from 3 dams. Two-way ANOVA. *P<0.05 *vs.* RUPP-Blank. †P<0.05 *vs.* RUPP-plCSA-NPs. ‡P<0.05 *vs.* RUPP-plCSA-RNPs. §P<0.05 *vs.* Control-Blank. ||P<0.05 *vs.* Sham-Blank. Scale bar: 1 cm.

**
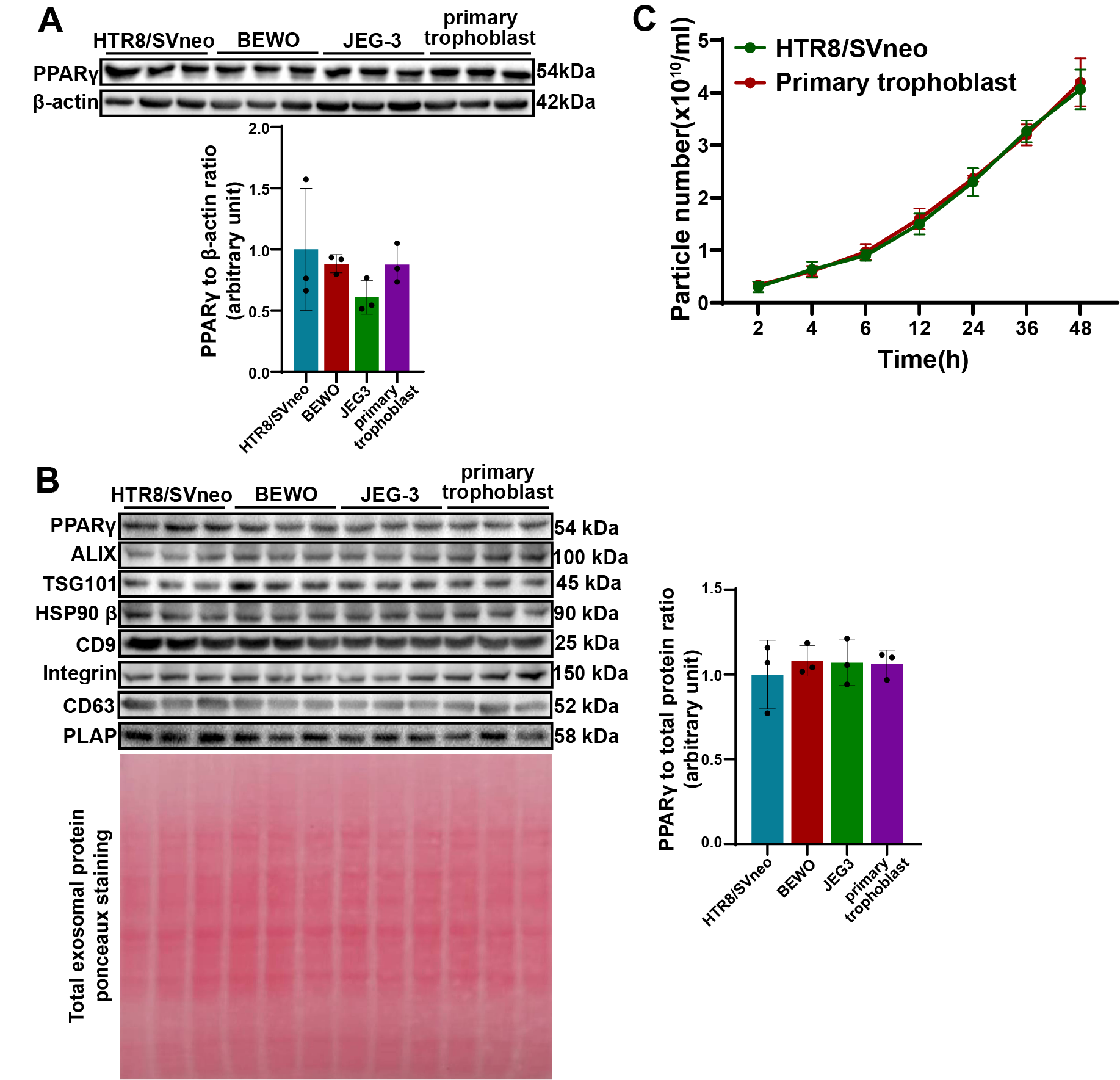
**

**Fig. S9: HTR8/SVneo cells mimic the abundance and secretion pattern of PPARγ in human term placenta-derived primary trophoblasts.**

**A.** Immunoblot analysis of PPARγ in HTR8/SVneo, BEWO, JEG3 and human term placenta-derived primary trophoblast cells. **B.** Immunoblot analysis of PPARγ, CD63, CD9, TSG101, ALIX, Integrin, PLAP and HSP90β in the exosomes of HTR8/SVneo, BEWO, JEG3 and human term placenta-derived primary trophoblast cells. **C.** Exosome secretion analysis of HTR8/SVneo and human term placenta-derived primary trophoblast cells via NTA. HTR8/SVneo cells (1x10^7^) and human term placenta-derived primary trophoblasts (1x10^7^) were simultaneously seeded into 100-mm tissue culture dishes separately in the corresponding complete culture medium. After 12 h (the cells were all attached to the wall), the medium was removed and replaced with 10 ml of the same medium prepared with exosome-depleted FBS. The supernatant was then collected 2 h, 4 h, 6 h, 12 h, 24 h, 36 h and 48 h later, and the exosomes were extracted. These exosomes were then subjected to quantification via NTA.

**Table S1**

**Characteristics of the exosomes**

| **Parameter** | **HTR8 trophoblasts** | | **Human placenta** | | **Mouse placenta** | |
| --- | --- | --- | --- | --- | --- | --- |
|  | **DOX-treated Tet-NC HTR8** | **DOX-treated Tet-shPPARγ HTR8** | **Normal**  **(Week 39)** | **FGR**  **(Week 39)** | **Sham**  **(GD 18.5)** | **RUPP**  **(GD 18.5)** |
| **Average count of particles per frame** | **243.7±53.01** | **234.7±59.35** | **79±20.81** | **172±6.557** | **223±97.08** | **202.3±43.47** |
| **Number of traced particles** | **2765±486.1** | **3123±794.9** | **1468±265.2** | **2133±562.9** | **3644±1529** | **3393±1127** |
| **Median (50×)** | **122.5±4.875** | **125±4.718** | **122±1.343** | **127.8±0.723** | **128.3±7.534** | **128.6±2.663** |
| **Peak diameter (nm)** | **130.1±3.305** | **132.6±4.206** | **125.8±2.095** | **130.6±0.781** | **133.3±7.927** | **137.5±3.345** |
| **Concentration (number per ml or mg)** | **1.43x10^10^±**  **5.49x10^9^** | **1.48x10^10^±**  **5.74x10^9^** | **2.88x10^8^±**  **7.63x10^7^** | **6.24x10^8^±**  **2.4x10^7^ *** | **8.05x10^8^±**  **3.58x10^8^** | **7.41x10^8^±**  **1.7x10^8^** |

The values are presented as the means±SDs. FGR: fetal growth restriction. The concentrations were analyzed via an unpaired two-tailed Student’s t test. * P<0.05 compared with the normal value in the human placenta. Exosomes in Tet-NC HTR8 and Tet-shPPARγ HTR8 cell supernatants were isolated after the cells were treated with 1 µg/ml DOX for 48 h; n=3 independent experiments with 3 independent preparations of exosomes. Placenta-derived exosomes were isolated from homogenates of the maternal face of human placentas and the labyrinthine zone of mouse placentas of the indicated gestational age (n=3 in the normal and FGR groups of human placentas, with 3 independent preparations of exosomes; n=3 in the sham and RUPP mouse placentas from 3 dams, with 3 independent preparations of exosomes).

**Table S2**

**Identification of PPARγ in human placenta-derived exosomes via proteomic analysis**

| **Reference** | **PepCount** | **Unique PepCount** | **MW** | **PI** |
| --- | --- | --- | --- | --- |
| **tr\|D2KUA6\|D2KUA6_HUMAN  Peroxisome proliferator-activated receptor gamma  OS=*Homo sapiens* OX=9606 GN=PPARG PE=2 SV=1** | **1** | **1** | **54680.48** | **6.19** |
| **tr\|Q53EW1\|Q53EW1_HUMAN  Peroxisome proliferator-activated receptor gamma (Fragment)  OS=*Homo sapiens* OX=9606 GN=PPARG PE=2 SV=1** | **1** | **1** | **54777.53** | **6.1** |
| **tr\|E9PFV2\|E9PFV2_HUMAN  Peroxisome proliferator-activated receptor gamma  OS=*Homo sapiens* OX=9606 GN=PPARG PE=1 SV=1** | **1** | **1** | **55360.27** | **6.19** |
| **tr\|A0A0S2Z4K5\|A0A0S2Z4K5_HUMAN  Peroxisome proliferator-activated receptor gamma (Fragment)  OS=*Homo sapiens* OX=9606 GN=PPARG PE=2 SV=1** | **1** | **1** | **30157.28** | **6.41** |
| **tr\|Q86 WD1\|Q86 WD1_HUMAN  Peroxisome proliferator-activated receptor gamma  OS=*Homo sapiens* OX=9606 GN=PPARG PE=2 SV=1** | **1** | **1** | **66632.42** | **5.29** |
| **sp\|P37231\|PPARG_HUMAN  Peroxisome proliferator-activated receptor gamma  OS=*Homo sapiens* OX=9606 GN=PPARG PE=1 SV=3** | **1** | **1** | **57619.51** | **5.61** |

The exosomes used for proteomic analysis consisted of a mixture of 3 biological samples isolated from 39 normal human placentas at 3 different ages.

**Table S3**

**Clinical characteristics of the participants**

| **Maternal characteristics** | **Normal (n=35)** | **FGR (n=35)** | **P value** | **95% CI** |
| --- | --- | --- | --- | --- |
| **Age (years)** | **28.2±4.093** | **26.86±2.415** | **0.0992** | **-2.946 to 0.2601** |
| **Prepregnancy BMI** | **20.73±2.205** | **21.33±1.768** | **0.2165** | **-0.3573 to 1.549** |
| **Uterine height (cm)** | **33.94±1.644** | **31.94±2.326** | **<0.0001*** | **-2.961 to -1.039** |
| **Abdominal circumference (cm)** | **102.3±5.079** | **93.2±6.548** | **<0.0001*** | **-11.88 to -6.291** |
| **GA at delivery (days)** | **276.7±5.57** | **266.1±6.67** | **<0.0001*** | **-13.59 to -7.726** |
| **Parity** | **2.171±1.403** | **1.886±1.323** | **0.384** | **-0.9364 to 0.3649** |
| **Neonatal characteristics** |  |  |  |  |
| **Body length (cm)** | **49.66±1.162** | **45.66±1.679** | **<0.0001*** | **-4.689 to -3.311** |
| **Birth weight (g)** | **3359±335.3** | **2285±186.5** | **<0.0001*** | **-1204 to -945.2** |
| **Head circumference (cm)** | **34.70±0.8062** | **31.33±2.292** | **<0.0001*** | **-4.187 to -2.549** |
| **Chest circumference (cm)** | **33.04±1.308** | **28.76±1.344** | **<0.0001*** | **-4.915 to -3.641** |
| **Abdominal circumference (cm)** | **32.83±0.9848** | **28.94±1.508** | **<0.0001*** | **-4.491 to -3.276** |
| **Sex (number, ratio)** | **Male (15, 42.86%)**  **Female (20, 57.14%)** | **Male (11, 31.43%)**  **Female (24,68.57%)** | **0.3224** |  |
| **NICU occupancy number (ratio)** | **0 (0%)** | **9 (25.7%)** | **0.0022*** |  |
| **Apgar 1 min** | **9.857±0.43** | **9.314±0.6761** | **0.0002*** | **-0.8131 to -0.2726** |
| **Appendants** |  |  |  |  |
| **Placenta weight** | **547.7±63.39** | **465.7±88.36** | **<0.0001*** | **-118.7 to -45.32** |
| **UCL (cm)** | **60.6±5.632** | **54.31±7.066** | **0.0001*** | **-9.333 to -3.238** |
| **AFV (ml)** | **575.4±104.6** | **429.1±196.8** | **0.0002*** | **-221.5 to -71.1** |

CI: confidence interval, BMI: body mass index (weight/height^2^), GA: gestational age, NICU: neonatal intensive care unit, UCL: umbilical cord length, AFV: amniotic fluid volume. All variables except sex and NICU occupancy number were analyzed via unpaired two-tailed Student’s t test. Pearson’s x^2^ test was used for analysis of sex, and the NICU occupancy number was determined via Fisher’s exact test. The values are presented as the means±SDs. *: Statistically significant difference between the normal and FGR groups.

**Table S4**

**Clinical characteristics of the second-trimester participants**

| **Maternal characteristics** | | **Neonatal characteristics** | |
| --- | --- | --- | --- |
| **Age (years)** | **25.5±1.871** | **Body length (cm)** | **29.17±9.538** |
| **Prepregnancy BMI** | **21.87±1.425** | **Birth weight (g)** | **505.5±266** |
| **Uterine height (cm)** | **19.33±5.502** | **Head circumference (cm)** | **185.3±60.27** |
| **Abdominal circumference (cm)** | **80±4.243** | **Abdominal circumference (cm)** | **171.8±62.38** |
| **GA at delivery (days)** | **155±36.09** | **Sex (number, ratio)** | **Male (3), female (3)** |
| **Parity** | **2±1.095** |  |  |

BMI: body mass index (weight/height^2^), GA: gestational age.

**Table S5**

**Information on the primers used for qRT‒PCR**

| **Gene** | **Sense (5'→3')** | **Antisense (5'→3')** |
| --- | --- | --- |
| ***PPARG*** | **GCCTGCATCTCCACCTTATTA** | **ATCTCCACAGACACGACATTC** |
| ***β-actin*** | **TGGCACCCAGCACAATGAA** | **CTAAGTCATAGTCCGCCTAGAAGCA** |
| ***Arid5b*** | **GCACCATCTTCACAACGAAC** | **GCTGCCACCTTCTTATCTTGC** |
| ***Htr2c*** | **CTAATTGGCCTATTGGTTTGGCA** | **CGGGAATTGAAACAAGCGTCC** |
| ***Rorb*** | **TGCTGAAATTCCTCCGAGA** | **TCTTTGTGCTTTAGTTTTGGCTA** |
| ***Pank2*** | **TAAAGCTCCACTTCACTTCTCC** | **TGCCCACAAGACATTACCTC** |
| ***Rplp0*** | **CCGCTACTGTTTACTTTGTGTTG** | **CAGACTAGCTATGGCACCAA** |

**Supplemental Video**

Resonant scan of a mouse placenta 24 h after injection of PKH67-labeled mouse placenta-derived exosomes.
